# Chorismate mutase and isochorismatase, two potential effectors of the migratory nematode *Hirschmanniella oryzae*, increase host susceptibility by manipulating secondary metabolite content of rice

**DOI:** 10.1111/mpp.13003

**Published:** 2020-10-20

**Authors:** Lander Bauters, Tina Kyndt, Tim De Meyer, Kris Morreel, Wout Boerjan, Hannes Lefevere, Godelieve Gheysen

**Affiliations:** ^1^ Department of Biotechnology Faculty of Bioscience Engineering Ghent University Ghent Belgium; ^2^ Department of Data Analysis and Mathematical Modelling Faculty of Bioscience Engineering Ghent University Ghent Belgium; ^3^ VIB‐UGent Center for Plant Systems Biology Ghent Belgium; ^4^ Department of Plant Biotechnology and Bioinformatics Faculty of Sciences Ghent University Ghent Belgium

**Keywords:** chorismate mutase, defense, *Hirschmanniella oryzae*, isochorismatase, nematode, *Oryza sativa*

## Abstract

*Hirschmanniella oryzae* is one of the most devastating nematodes on rice, leading to substantial yield losses. Effector proteins aid the nematode during the infection process by subduing plant defence responses. In this research we characterized two potential *H. oryzae* effector proteins, chorismate mutase (HoCM) and isochorismatase (HoICM), and investigated their enzymatic activity and their role in plant immunity. Both HoCM and HoICM proved to be enzymatically active in complementation tests in mutant *Escherichia coli* strains. Infection success by the migratory nematode *H. oryzae* was significantly higher in transgenic rice lines constitutively expressing *HoCM* or *HoICM*. Expression of *HoCM*, but not *HoICM*, increased rice susceptibility against the sedentary nematode *Meloidogyne graminicola* also. Transcriptome and metabolome analyses indicated reductions in secondary metabolites in the transgenic rice plants expressing the potential nematode effectors. The results presented here demonstrate that both HoCM and HoICM suppress the host immune system and that this may be accomplished by lowering secondary metabolite levels in the plant.

## Graphical abstract

Chorismate mutase and isochorismatase from the plant parasitic nematode *Hirschmanniella oryzae* are able to decrease immunity of rice by interfering with the phenylpropanoid pathway.

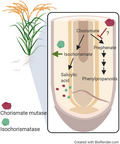

## 1 INTRODUCTION

*Hirschmanniella oryzae* is one of the most devastating pests in rice fields worldwide. Because *H. oryzae* is well adapted to flooded conditions, it is a major problem in flooded rice ecosystems (Babatola, 1981). This migratory nematode penetrates the root and migrates through the aerenchyma, completing its life cycle in about 33 days (Karakas, 2004). Plant‐parasitic nematodes are equipped with an arsenal of cell wall‐modifying proteins that are secreted through the stylet into the host tissue to hydrolyse cell walls (Davis et al., 2011). Sedentary nematodes establish a feeding site, whereby it is important to remain undetected by the plant to prevent an adequate defence response. Effectors are secreted by this type of nematode to alter plant cell metabolism to their advantage and to attenuate the defence mechanisms of the host (Postma et al., 2012; Hewezi and Baum, 2013; Jaouannet et al., 2013). Migratory nematodes, like *H. oryzae*, stay mobile during their entire life cycle. They keep on migrating through the root whilst feeding. Although this lifestyle might enable them to outrun local plant defences and effector proteins inhibiting plant defence might seem unnecessary, several potential effector proteins have been predicted from the transcriptome of plant‐parasitic migratory nematodes (Haegeman et al., 2011; Nicol et al., 2012; Bauters et al., 2014). This implies that migratory nematode species also invest energy in attenuating the defence responses of their host.

The plant hormone salicylic acid (SA) plays an important role as a signalling molecule during the defence reaction on pathogen infection. SA can be produced through two distinct pathways starting from chorismate, the final product of the shikimate pathway: the isochorismate synthase (ICS) pathway and the phenylalanine ammonia lyase (PAL) pathway (leading to SA and phenylpropanoids). Several chorismate‐derived compounds play a role in plant physiology and the defence system, for example, SA, auxin, lignin, and flavonoids. Changes in concentrations of these compounds can affect the plant defence against pathogen infection (Jones et al., 2007; Nahar et al., 2012). Interestingly, transcripts encoding chorismate mutase and isochorismatase were detected in the transcriptome of *H. oryzae* (Bauters et al., 2014). Both enzymes can interfere in the host production of salicylic acid and/or phenylpropanoids. Chorismate mutase catalyses the conversion of chorismate to prephenate. This enzyme is present in plants and bacteria but had not been reported in animals until its discovery in *Meloidogyne javanica* (Lambert et al., 1999). Since then it has been reported in several other plant‐parasitic nematodes (Jones et al., 2003; Huang et al., 2005; Vanholme et al., 2009). It has been shown that chorismate mutase, secreted by plant‐pathogenic organisms, is able to alter plant cell development by impairing the development of lateral roots and vascular tissue (Doyle and Lambert, 2003). More recent research provided experimental evidence that a secreted chorismate mutase originating from *Ustilago maydis* reduced SA levels in host plants (Djamei et al., 2011). A decrease in SA content was also observed in tobacco leaves transiently expressing a chorismate mutase from *Meloidogyne incognita* (*Mi‐CM3*) upon infection by the oomycete *Phytophthora capsici*. In addition, constitutive expression of *Mi‐CM3* increased susceptibility of tobacco plants to *M. incognita* infection (Wang et al., 2018). The gene encoding isochorismatase has not been characterized in nematodes before, but it was reported to be present in the genome of *Meloidogyne hapla* and the transcriptome of *Rotylenchulus reniformis* (Opperman et al., 2008; Wubben et al., 2010). Moreover, isochorismatase was detected in the secretome of plant‐pathogenic fungi, but not in nonpathogenic species (Soanes et al., 2008) and it was reported to reduce SA content in plants on infection. Isochorismatase converts isochorismate to 2,3‐dihydroxy‐2,3‐dihydrobenzoate and pyruvate. Isochorismate is an intermediate in the biosynthesis of SA, hence isochorismatase is capable of reducing the pool of isochorismate available for SA synthesis (Liu et al., 2014).

In this research, the genetic structure and protein properties of chorismate mutase (*HoCM*) and isochorismatase (*HoICM*) isolated from *H. oryzae* are described. Their activity is demonstrated by complementation of chorismate mutase (*CM*) and isochorismatase (*ICM*) deficient *Escherichia coli* strains, and infection assays with *H. oryzae* on transgenic plants showed that both proteins increase susceptibility of the host plant to nematode infection. Moreover, *HoCM,* but not *HoICM* expression, had an effect on infection with the sedentary nematode *Meloidogyne graminicola*. Finally, RNA‐Seq performed on rice plants expressing *HoICM* or *HoCM* revealed that both proteins interfere with secondary metabolic pathways, a result supported by metabolome analysis.

## 2 RESULTS

### 2.1 Genetic structure of *HoCM* and *HoICM*

**FIGURE 1:**
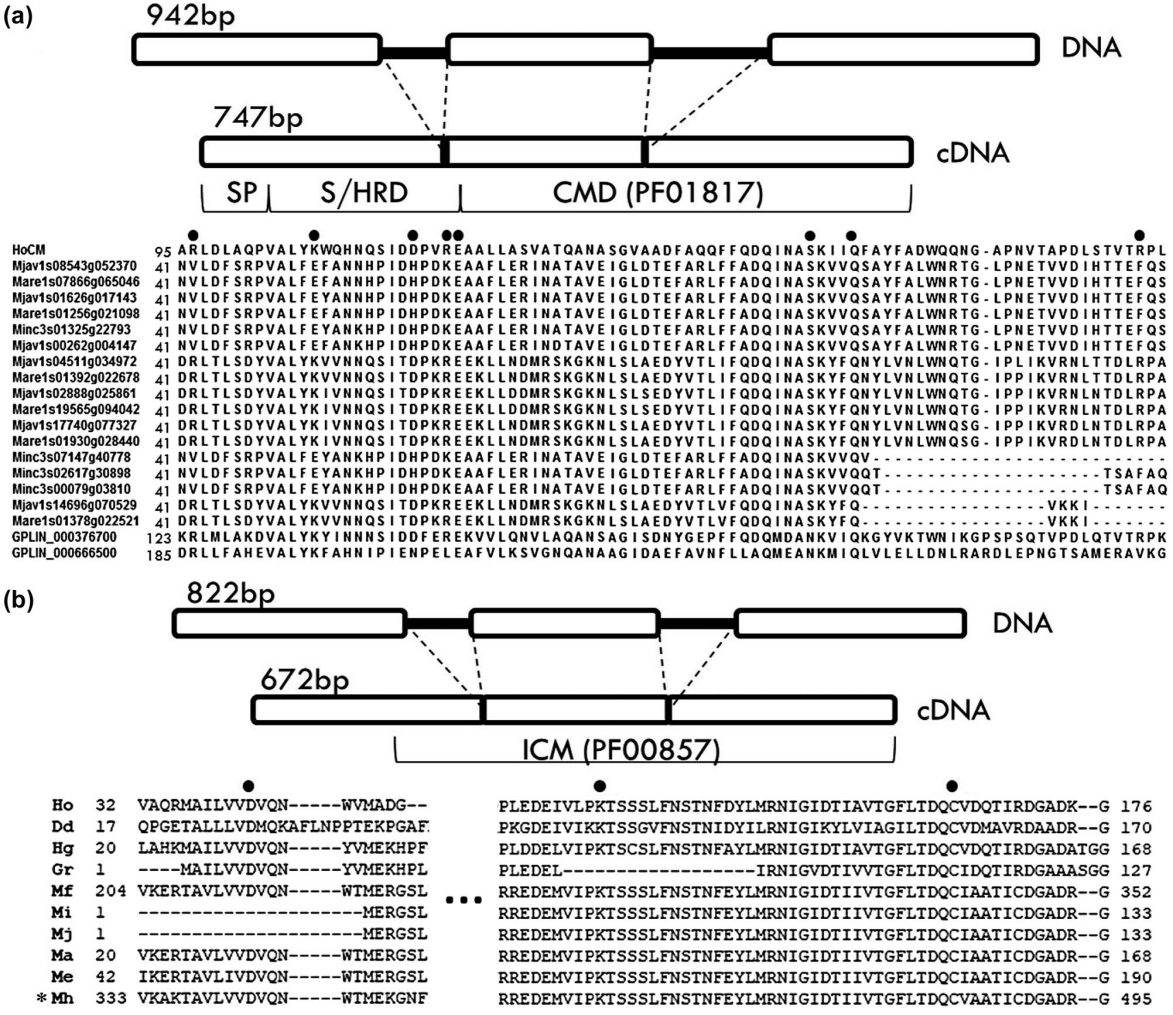
Gene structure and partial protein sequence of *HoCM* and *HoICM*. (a) *HoCM* has a total length of 942 base pairs at the genomic level, containing two small introns. The protein consists of three different domains: an N‐terminal signal peptide (SP), a serine/histidine‐rich domain (S/HRD), and a chorismate mutase domain (CMD) (PF01817). The alignment shows the conserved catalytic residues in several plant‐parasitic nematode species (Mjav, *Meloidogyne javanica*; Mare, *Meloidogyne arenaria*; Minc, *Meloidogyne incognita*; GPLIN, *Globodera pallida*). Eight conserved putative catalytic residues characterized in chorismate mutase of *Mycobacterium tuberculosis* and/or *Escherichia coli* are marked with filled dots. (b) *HoICM* is 822 bp long and contains two small introns. It contains an isochorismatase domain (PF00857), but no predicted N‐terminal signal peptide. The three putative catalytic residues are marked with filled dots. Sequence names correspond to the protein sequences encoded by different nematodes, downloaded from the Wormbase Parasite database (Ho, *Hirschmanniella oryzae*, ALI53582; Dd, *Ditylenchus destructor*, Dd_10059; Hg, *Heterodera glycines*, Hetgly.G000001356; Gr, *Globodera rostochiensis*, GROS_g01640.t1; Mf, *Meloidogyne floridiensis*, scf7180000418840.g2997; Mi, *M. incognita*, Minc3s04290g35947; Mj, *M. javanica*, M.javanica_Scaff754g009582; Ma, *M. arenaria*, M.arenaria_Scaff10008g072685; Me, *Meloidogyne enterolobii*, scaffold11435_cov287.g14711; Mh, *Meloidogyne hapla*, MhA1_Contig1915.frz3.gene2). *Sequence adjusted (part of intron used as exon) due to probable mistake in gene prediction

The genomic sequence of chorismate mutase (*HoCM,* KP297892) consists of 942 nucleotides from start to stop codon, and contains two introns of 72 and 123 nucleotides. The isochorismatase gene (*HoICM,* KP297893) consists of 822 nucleotides from start to stop codon and includes two introns of 69 and 81 base pairs (Figure 1a,b). All exon/intron transitions contain the consensus splice site GT/AG, except for one *HoICM* noncanonical splice site GT/CG. The first 60 nucleotides of *HoCM* code for a secretion signal, targeting the encoded protein to the classical secretory pathway. Expression in the secretory glands was shown by in situ hybridization in previous research (Bauters et al., 2014). No secretion signal was detected in *HoICM*. Several attempts were made to perform an in situ hybridization for *HoICM* transcripts, but all of them were negative or showed ambiguous results. *HoICM* was PCR‐amplified from genomic DNA extracted from a single nematode, three times, from an independent single hand‐picked nematode. *HoICM* could be amplified in all three experiments, supporting that this is an endogenous *H. oryzae* gene rather than being derived from any contaminating material. The fact that this type of isochorismatase (ICM) is specific to plant‐parasitic species (Bauters et al., 2014) and that it is secreted by filamentous pathogens through a nonclassical secreted pathway (Liu et al., 2014) points towards an effector function in nematodes as well.

### 2.2 Important catalytic residues are conserved in both HoCM and HoICM

The HoCM protein consists of three domains, including the signal peptide. The N‐terminal domain of the mature protein has no similarities with other domains in public databases, but it has two motifs, rich in serine and histidine, respectively, hence the name serine/histidine‐rich domain (S/HRD). The C‐terminal part is similar to the Pfam chorismate mutase (CM) type II domain (PF01817) (CMD). Alignment of HoCM and other CM sequences deduced from the genomes of cyst and root‐knot nematodes showed conservation of catalytic residues as initially characterized in *E. coli* and *Mycobacterium tuberculosis* (Lee et al., 1995; Ökvist et al., 2006). The eight conserved catalytic residues are highlighted in Figure 1a. All residues are conserved in *H. oryzae*, but far less in cyst and root‐knot nematodes.

An ICM domain (PF00857) is predicted in the C‐terminal part of the HoICM protein. Homologous sequences are present in the expressed sequence tag (EST) database of *R. reniformis*, *Radopholus similis*, and *Heterodera glycines*. No homologues were found in the genome of *Globodera pallida*, but several homologous sequences were retrieved from the genomes of different *Meloidogyne* spp. and *Globodera  rostochiensis*. Catalytic residues in ICMs have been identified in *Oleispira antarctica* and *Pseudomonas aeruginosa*, and are conserved in *H. oryzae* and other plant‐parasitic nematodes (Figure 1b) (Parsons et al., 2003; Goral et al., 2012).

### 2.3 Predicting the protein structure of HoCM and HoICM

**FIGURE 2:**
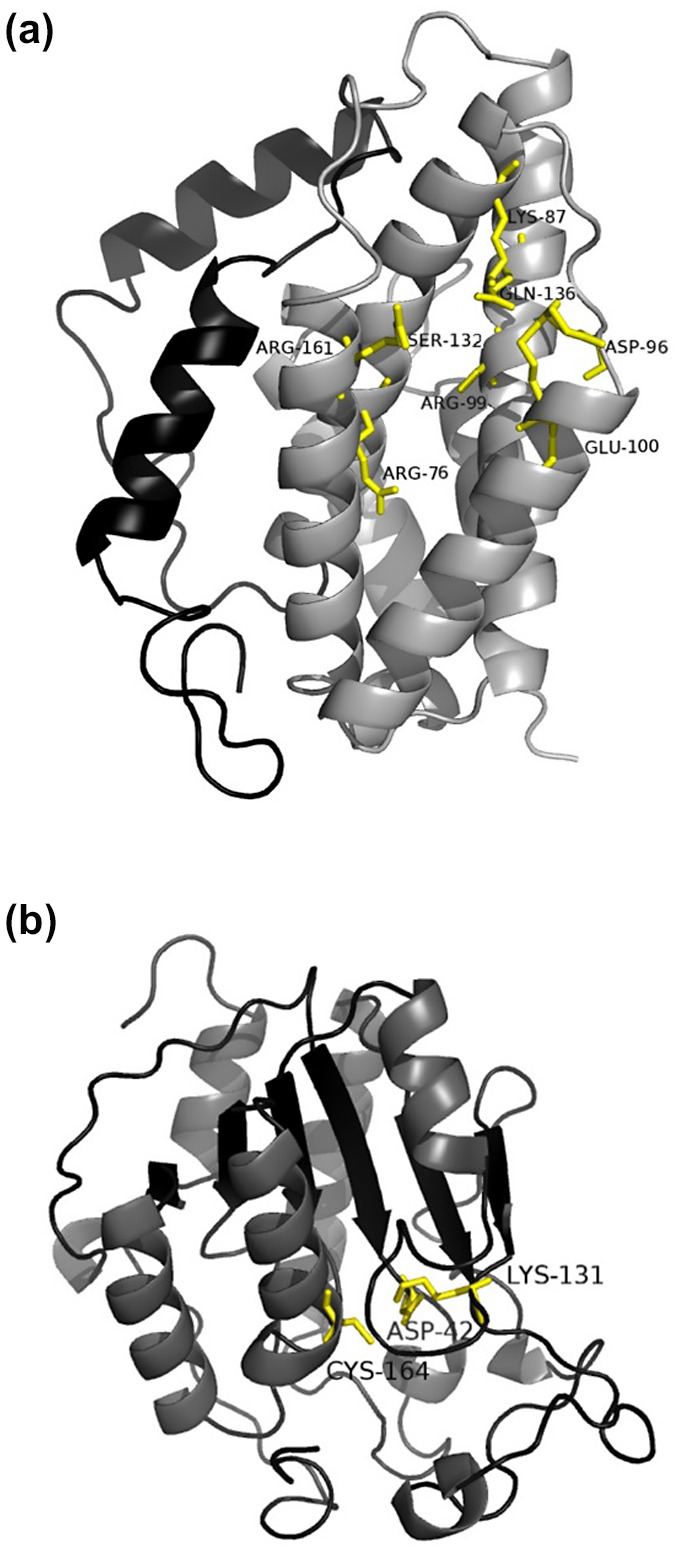
Predicted tertiary structure of HoCM and HoICM. (a) HoCM. The structure of the mature protein is shown (without secretion signal). It was constructed using homology modelling with chorismate mutase models of *Mycobacterium tuberculosis* and *Yersinia pestis* as templates. The C‐terminal chorismate mutase domain (grey) consists of two large α‐helices in the middle, surrounded by four smaller α‐helices. The N‐terminal part (black) is predicted to have two α‐helices. Eight catalytic residues are shown as stick models. (b) HoICM. The six‐stranded parallel β‐sheet (black) is in the centre of the molecule, surrounded by three α‐helices at one side and two at the other side. At the bottom (three) and the top left corner (one) in this view of the molecule there are four 3_10_‐helices. The catalytic triad (Cys164‐ Asp42‐Lys131) is labelled

CMs are classified into two main groups according to their structure: the AroH and AroQ classes. Proteins of the rare AroH class, represented by the CM of *Bacillus subtilis*, have both α‐helices and β‐sheets in their structure (Chook et al., 1993). On the other hand, proteins of the AroQ class are more abundant and are represented by the CM structure of *E. coli*, containing only α‐helices (Lee et al., 1995). Secondary and tertiary structure predictions showed that HoCM is composed of α‐helices and loops, without β‐sheets, indicating that HoCM is a member of the AroQ class. A 3D model was constructed using Phyre2 with the CM structures of *M. tuberculosis* (PDB code 2FP1) and *Yersinia pestis* (2GBB) as a template. The predicted tertiary structure of HoCM is shown in Figure 2a. The CM domain of HoCM has the same predicted topology as the global structure of an AroQ_γ_ protein, which consists of six α‐helices connected by loops. The S/HRD region (N‐terminal) of HoCM lacked homology and was modelled ab initio with only 72% of the residues modelled with over 90% confidence.

ICM‐like hydrolases (ILH) are a large family of enzymes divided into several structural subgroups by the Structural Classification of Proteins (SCOP release 2.03), that is, YecD, *N*‐carbamoylsarcosine amidohydrolase, phenazine biosynthesis protein (PhzD), pyrazinamidase/nicotinamidase, ribonuclease MAR1, and YcaC. These proteins belong to the class of “alpha‐beta‐alpha sandwich” according to the CATH (Class Architecture Topology/fold Homologous superfamily) structural classification, adopting a Rossman fold. The 3D model of HoICM was constructed using six different ICM structures (PDB code: 1NF9, 1NBA, 2FQ1, 3TB4, 3IRV, and 3KL2), creating a model for which 95% of the residues were modelled with more than 90% confidence with Phyre2. The model shows a molecule with a Rossman fold; a six‐stranded parallel β‐sheet flanked by three big α‐helices at one side and two at the other. Next to these α‐helices, there are four putative 3_10_‐helices present. The presumed catalytic triad is shown in Figure 2b. The catalytic centre where isochorismate can bind has been described in *Oleispira antarctica* and is conserved in other plant‐parasitic nematodes (PPNs) (Goral et al., 2012). PyMOL (v. 1.6) structural alignment assigns HoICM to the structural subgroup of the phenazine biosynthesis proteins (root mean square deviation of 0.437).

### 2.4 *HoCM* and *HoICM* complement *E. coli* mutants

**FIGURE 3:**
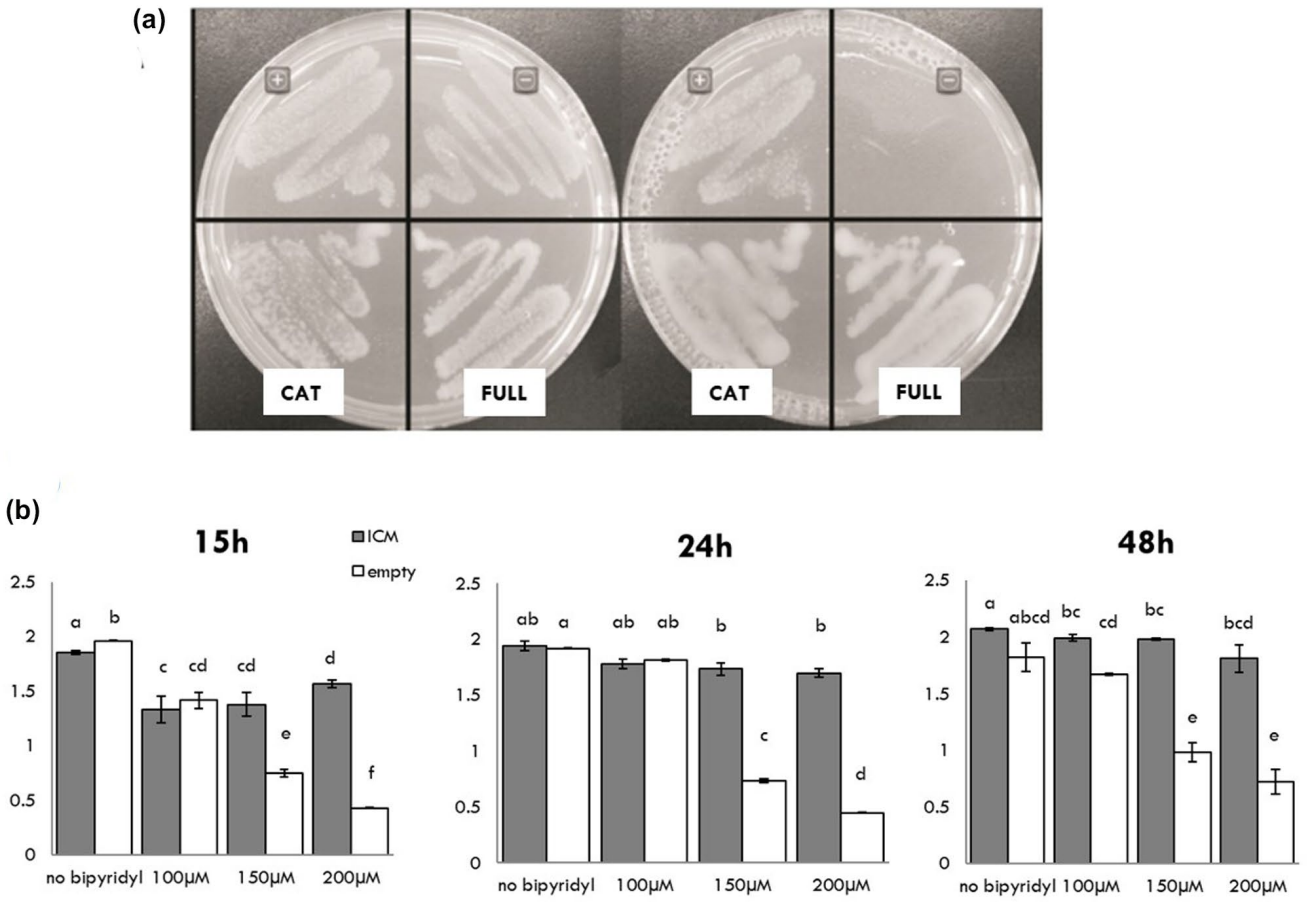
Complementation tests of HoCM and HoICM. (a) Complementation assay of the chorismate mutase (CM)‐deficient *Escherichia coli* strain KA12/pKIMP‐UAUC with different constructs of HoCM. The left Petri dish shows bacterial growth on M9cY medium supplemented with phenylalanine, the right one bacterial growth on M9cY medium without additions. +, positive control (CM of *Bacillus subtilis*); −, negative control (empty vector pQE30‐UA); Cat, catalytic domain of CM; Full, mature CM protein. (b) Complementation assay of *entB*‐mutated *E. coli* AN192. AN192 cells were complemented with an empty vector (pDEST17) (white) or HoICM (grey). Cells were grown in liquid medium with different concentrations of bipyridyl to create iron‐limiting conditions. Bacterial growth was observed by measuring optical density (*y* axis) at three different time points (an average was taken from four bacterial cultures). Treatments were compared with the control (no bipyridyl) and statistically analysed by a Mann–Whitney test. Each experiment was performed twice with similar results. Different letters above the graph indicate the significant differences between the different treatments

The activity of two different *HoCM* constructs was assessed by a complementation assay in a CM‐deficient *E. coli* strain (KA12/pKIMP‐UAUC) grown on dropout medium without phenylalanine. One construct contained the full *CM* coding sequence without the predicted signal peptide, the second only consisted of the catalytic region. The positive control was the *E. coli* mutant expressing a *B. subtilis CM*. The results confirmed CM activity of the HoCM protein (Figure 3a). The fact that mutants with both protein constructs grew equally well reveals that the S/HRD region is not necessary for CM activity.

The activity of HoICM was tested using an *ICM* (*entB*) deficient strain (AN192) incapable of producing the siderophore enterobactin, an iron chelator. The growth of AN192 and AN192 complemented with *HoICM* under iron‐limiting conditions, created by adding bipyridyl, was monitored by measuring optical density. Results showed that AN192 cells harbouring *HoICM* grew much better under iron‐limiting conditions compared to the empty vector control (Figure 3b). Fifteen hours postinoculation, there still was a significant difference (*p* value < .05) between bacteria growing under normal conditions and bacteria growing in iron‐limiting conditions, but after 24 hr the cells complemented with *HoICM* grew as well as the cells under conditions without bipyridyl. AN192 cells that carried the empty vector could not grow well under iron‐limiting conditions, the growth rate of this control remaining far below the growth rate of *HoICM*‐complemented cells under the two most stringent conditions (150 and 200 µM bipyridyl). This result proves that HoICM can complement the lack of ICM activity in the AN192 strain.

### 2.5 Rice lines expressing *HoCM* or *HoICM* are more susceptible to nematode infection

**FIGURE 4:**
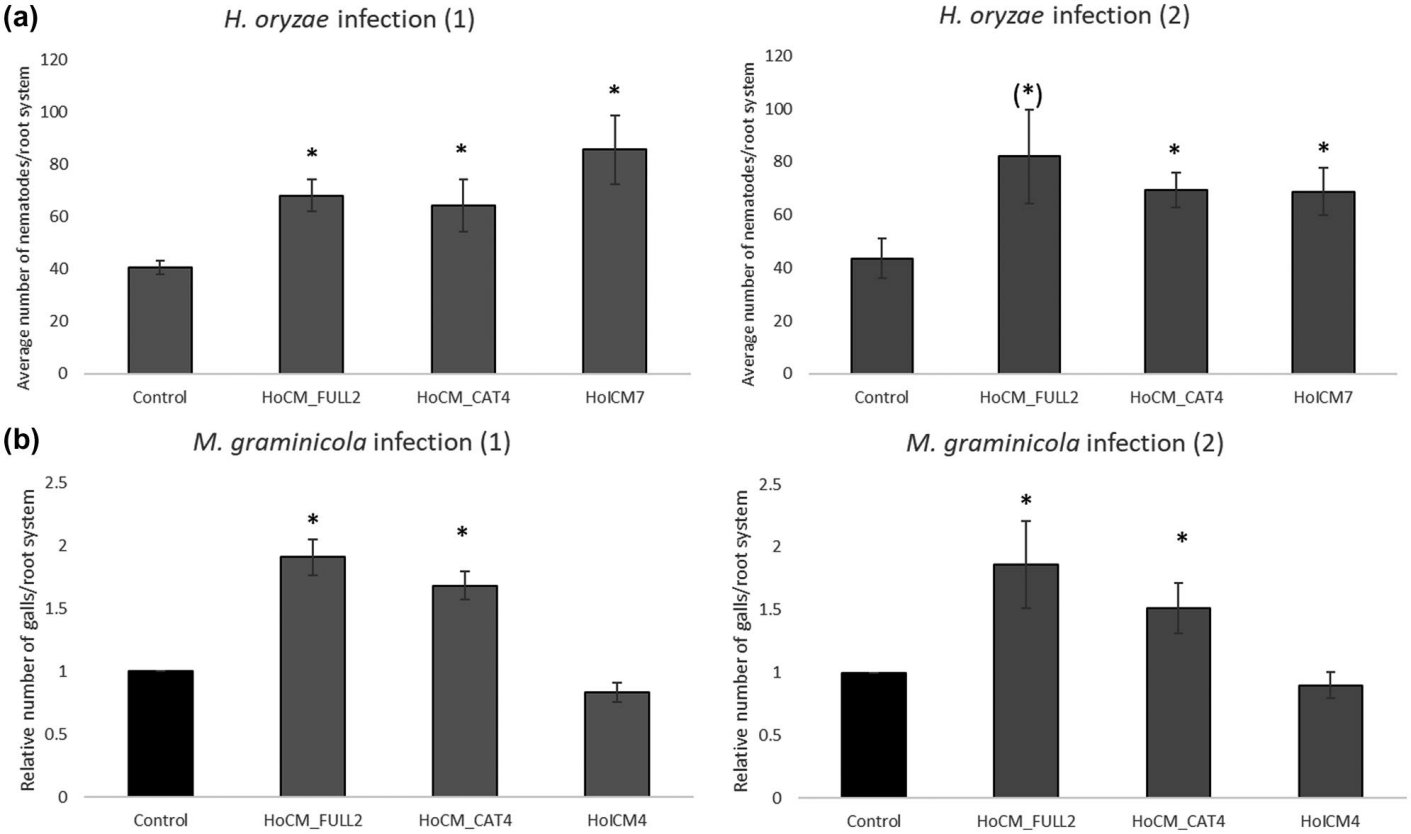
Effect of expression of HoICM, HoCM_FULL, and HoCM_CAT in rice on susceptibility against *Hirschmanniella oryzae* (a) and *Meloidogyne graminicola* (b). Bars represent the average number of nematodes (*H. oryzae*) or galls (*M. graminicola*) of eight infected plants per line. The number of galls was expressed relative to the control, which was set to 1. Asterisks indicate values significantly different from the control (empty vector) according to a nonparametric Mann–Whitney test (*p* < .05). (*) indicates *p* = .052. Error bars represent the standard error of the mean. Data for *M. graminicola* infection are expressed as relative values. The two repetitions of each experiment are shown. The infection was conducted on one line per construct

Transgenic rice lines constitutively expressing one of two versions of *HoCM* (*HoCM* without signal peptide (*HoCM_FULL*) or only the catalytic domain of *HoCM* (*HoCM_CAT*)) or *HoICM* were generated starting from *O. sativa* "Nipponbare." Lines transformed with a vector without insert (empty vector) were used as control. Five and two independent lines were obtained for *HoCM_CAT* and *HoCM_FULL*, respectively. For *HoICM*, eight independent lines were obtained, but only three of them produced enough viable seeds to perform further experiments. No clear phenotypes were observed between independent lines during the entire life cycle of the plant. The presence of the construct was verified at the genomic level by PCR with gene‐specific primers. Expression was validated by quantitative reverse transcription PCR (RT‐qPCR; see Table S1a,b). The transgenic lines were tested for their susceptibility to nematode infection (Figure 4). Constitutive expression of the nematode *HoCM* or *HoICM* enhanced susceptibility of the rice plants against *H. oryzae*. All three different constructs showed similar results. The transgenic lines were used in an infection assay with the sedentary root‐knot nematode *M. graminicola* as well. In contrast with the results obtained for *H. oryzae*, two lines of *HoICM*‐expressing rice plants showed no difference in *M. graminicola* infection levels with the control. On the other hand, both HoCM constructs enhanced susceptibility against *M. graminicola*, similar to the results obtained for *H. oryzae* (Figure 4).

### 2.6 Several genes involved in plant defence are down‐regulated in rice lines constitutively expressing the potential nematode effectors

RNA‐Seq was performed on one line per construct to study transcriptional changes in rice plants expressing *HoCM_CAT*, *HoCM_FULL*, or *HoICM*. Almost 150 million sequence fragments were generated. Almost 95% of all reads could be mapped to the rice genome (IRGSP‐1.0, see Table S2).

Expression levels of all genes in the transgenic lines were compared to those in the line transformed with the empty vector. Differentially expressed genes (DEGs) with a false discovery rate (FDR) <0.05 were compared using blastp to protein data for Viridiplantae available in the SwissProt database for annotation purposes. Compared to the empty vector line, the transgenic line expressing *HoCM_FULL* had 55 DEGs (13 up‐regulated, 42 down‐regulated). When *HoCM_CAT* was expressed, 196 DEGs were detected (67 up, 129 down), and 105 DEGs (36 up, 69 down) were found in plants expressing *HoICM*. Approximately half of these genes had a significant (bit‐score > 50) hit when compared with blast against the SwissProt database. These annotated genes were classified into six groups according to their putative function (File S1). Expression of 20 genes was down‐regulated in the transgenic plants expressing any of the three constructs, among which were two glutathione *S*‐transferases, a mono‐oxygenase, and geranylgeranyl pyrophosphate synthase. A nonprotein‐coding transcript was up‐regulated in all three constructs. Nine genes had the same trend in transgenic plants with either CM construct, among which were a down‐regulation of genes involved in steroid biosynthesis and auxin transport. It is interesting to note that many genes involved in signalling, stress response, and secondary metabolite production were differentially expressed upon in planta expression of both nematode genes. An overview of the significantly differentially regulated genes can be found in File S1.

The reliability of the RNA‐Seq results was validated by evaluating the expression of 12 randomly chosen DEGs by RT‐qPCR. Eleven of the genes confirmed RNA‐Seq results; only one gene showed a contrasting expression pattern: the gene coding for histone H2B.3 was significantly up‐regulated according to the RNA‐Seq data (log_2_FC = 1.6) in plants expressing *HoCM_CAT*, while it was down‐regulated according to the RT‐qPCR data. The expression of two genes, coding for glutathione *S*‐transferase and a receptor‐like protein kinase, was so strongly down‐regulated that it was undetectable by RT‐qPCR in the transgenic lines (see Figure S1). The clear correspondence with RT‐qPCR results for these 12 genes (*p* = .006, binomial test) supports the reliability of the generated RNA‐Seq data.

### 2.7 Gene ontology enrichment analysis shows a decrease in defence compound biosynthesis terms

**FIGURE 5:**
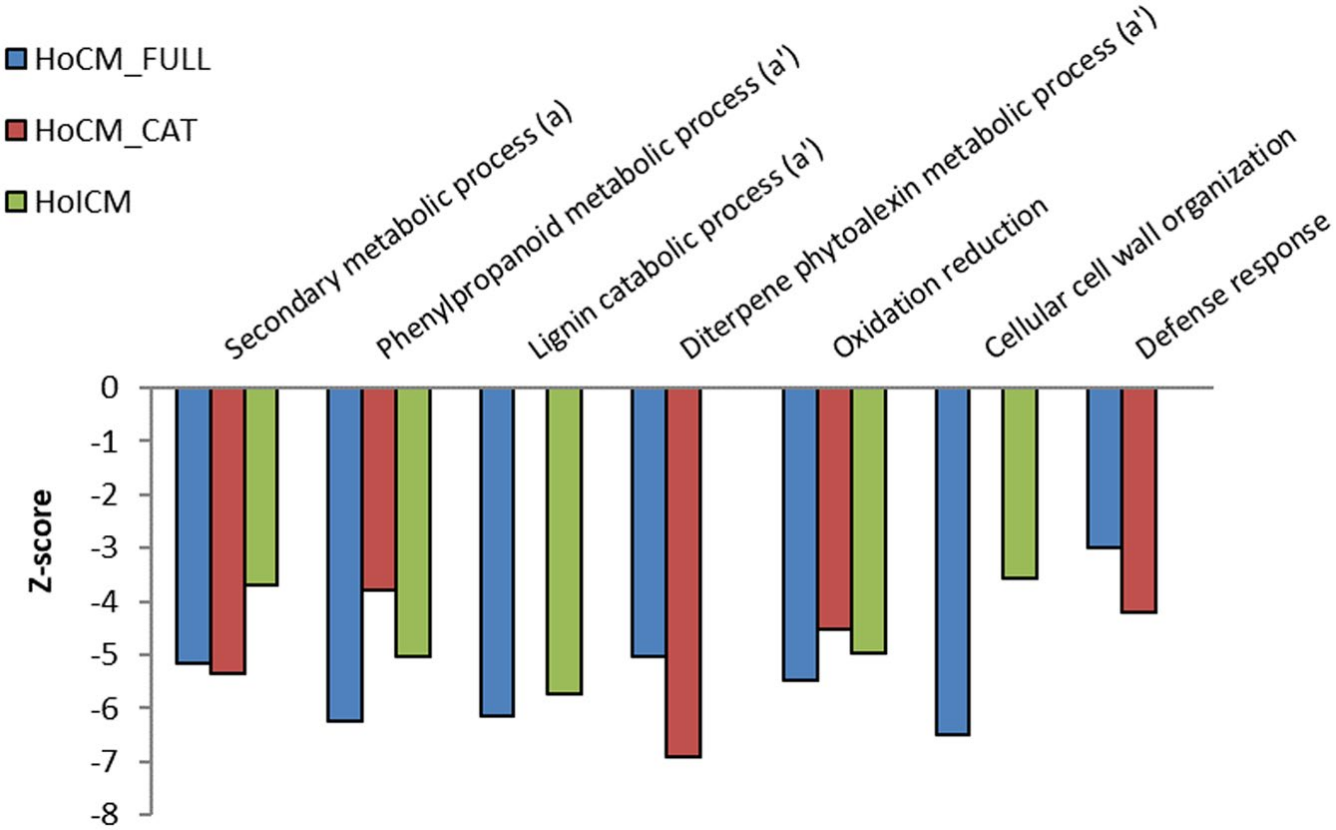
Gene ontology (GO) term enrichment analysis, showing the significantly enriched GOterms in the category “Biological process”. GO terms for which at least one of the three constructs resulted in plant transcripts with significant difference with an absolute *Z*‐score (*y* axis) value of 4 are shown in the graph. a and aʹ indicate an ancestor term (a) with its child terms (aʹ). All GO terms represented in this graph are significantly less abundant in the transgenic lines (*Z*‐score < 0) compared to control plants (empty vector line). Nonsignificant data are not shown in the graph

Parametric analysis of gene set enrichment (PAGE) was conducted using the log_2_ fold changes of all genes as input. All levels of gene ontology (GO) in the category “Biological Process” were considered, with a significance level of 0.05 (FDR). GO terms with a calculated absolute *Z*‐score higher than 4 for at least one of the three constructs are visualized in Figure 5. The expression of each different construct induces a repression of the “secondary metabolic process” GO in rice. Looking at the child terms of this GO term, the repression was due to a reduction in the GO term “Phenylpropanoid metabolic process”. The GO term “Lignin catabolic process” is reduced in *HoCM_FULL‐* and *HoICM*‐expressing plants. “Diterpene phytoalexin metabolic process” is suppressed in plants expressing *HoCM*. Rice plants expressing either one of the *HoCM* constructs are also repressed in the general “Defense response”, while all three lines are reduced in “Oxidation reduction”.

### 2.8 Metabolome analysis suggests that HoCM down‐regulates phenylpropanoid biosynthesis

No differences in SA content in shoots or roots were detected in transgenic lines compared to the control (Figure S2) using the protocol described by Haeck et al. (2018). To obtain more insight into the effects of either *ICM* or *CM* on phenolic metabolism in rice roots, a comparative liquid chromatography‐mass spectrometry (LC‐MS, see Experimental procedures) profiling was performed on the methanol extracts from *HoICM*‐expressing plants and the control line (referred to as the *HoICM* set) and from *HoCM_FULL*, *HoCM_CAT*, and the control line (referred to as the *HoCM* set). Taking both sets together, approximately 13,884 compounds (as estimated by the number of *m*/*z* feature groups) were profiled. The abundances of these compounds were analysed by applying an analysis of variance (ANOVA) model separately for each set, and correcting for the large number of tests by calculating the FDR (α = .01). The numbers of so‐obtained differential compounds for the *HoICM* and *HoCM* sets were 53 (Table S3) and 17 (Table S4), respectively. In the *HoCM* set, 1 and 16 compounds displayed higher and lower levels in the transgenic lines as compared to the control lines. However, all of them were of low abundance, hindering the recording of good‐quality MS/MS spectra for structural elucidation (Table S5). For the *HoICM* set, 7 and 46 compounds showed higher and lower levels in the transgenic lines as compared to the control lines, respectively. The most differentially accumulating compound in the *HoICM* line was structurally characterized as 1‐*O*‐ethyl‐6‐*O*‐glycoloyl hexose. In addition, another of the top accumulating compounds was very similar in the MS/MS spectrum and was identified as (1‐*O*‐ethyl)hexosyl(6‐>)ethylene glycol glycolate. Both compounds indicate that expression of *HoICM* leads to the accumulation of glycolic acid. In addition, many of the compounds that showed diminished levels in the *HoICM* lines were probably fatty acid‐derived molecules that contained a glycine moiety based on the chemical formula and the detection of glycine‐representing MS/MS product ions (Table S5 and Figures S3 and S4).

Because it proved to be difficult to characterize the unknown compounds that were statistically different in abundance between treatment and control, as an alternative approach only those metabolites that could be matched with a known compound were taken into consideration. To increase confidence in the proposed matches, only compounds with a fragmentation score higher than 0.8 were kept. This pipeline resulted in a list of only 17 compounds, 16 of which were linked to the phenylpropanoid pathway. Although not significantly different, for *HoCM*‐expressing lines 13 compounds had lower levels in the two independent lines per construct, compared to the control, suggesting down‐regulation of the phenylpropanoid pathway. A two‐way ANOVA using “relative abundance of each component” as variable and “control vs transgenic” and “independent line” as fixed and random factors, respectively, was conducted. A general lower abundance of compounds from the phenylpropanoid pathway in *HoCM*‐expressing lines (*p* = .008) was observed without any effect of the “independent line” factor (*p* = .5). Because there was an interaction effect between the fixed and the random factor for *HoICM*‐expressing plants, no clear conclusion could be drawn (see Table 1).

**TABLE 1:** Relative values for characterized secondary metabolite abundance in rice lines expressing HoCM or HoICM compared to the control line

	Compound ID	HoCM_FULL2	HoCM_CAT2	HoCM_CAT3	HoICM1	HoICM4
Phenylpropanoid	Tricin(4'‐*O*‐8)G‐5/7‐*O*‐hexoside 3	0.4023	0.2582	1.1936	4.6489	1.2003
Phenylpropanoid	Vanillic acid 1	0.3996	0.5069	0.4375	1.2130	0.4053
Phenylpropanoid	Syringic acid 4‐*O*‐hexoside	0.3837	0.4415	0.4575	1.2970	0.7224
Phenylpropanoid	Vanilloyl hexose	0.1163	0.1129	0.1869	0.6548	0.6579
Phenylpropanoid	4‐Hydroxybenzoic acid	0.3824	0.4140	0.6249	2.1176	1.6102
Phenylpropanoid	Feruloyl quinate 1	0.3513	0.6880	0.5078	1.2792	0.5786
Phenylpropanoid	Ferulic acid 4‐*O*‐hexoside 2 (‐dehydrated hexose)	1.0309	0.9846	0.5018	1.4663	0.5709
Phenylpropanoid	Sinapoyl hexose	0.4679	0.2185	0.6017	1.0430	1.0901
Phenylpropanoid	*p*‐Coumaric acid	0.3871	0.4913	0.6304	1.7322	1.0924
Phenylpropanoid	Caffeoyl shikimic acid 1	0.3424	0.6480	0.5111	0.5509	0.3112
Phenylpropanoid	*t*‐Ferulic acid	0.3413	0.3526	0.3254	0.6086	0.0763
Phenylpropanoid	*m*‐Hydroxycinnamic acid	0.3302	0.4654	0.5502	1.7059	1.1033
Phenylpropanoid	G(red8‐5)G + hexose	0.2210	0.8074	0.6627	0.6986	0.2126
Phenylpropanoid	G(8‐8)G hexoside 1	0.3728	0.9640	0.5749	0.5736	0.2960
Phenylpropanoid	G(red8‐5)G 8‐*O*‐hexoside	0.3414	0.3420	0.6841	3.3869	0.7431
Phenylpropanoid	G(8‐5)G [−H2O] 2	0.3202	0.2526	0.5464	1.1624	0.3864
		*p* = .008			*p* = .865	
Jasmonate	12‐Hydroxy jasmonic acid sulphate	0.4016	0.5912	0.4852	0.8332	0.7970

Note

Compounds that have a lower abundance compared to the control have a value < 1. *p* values show the result of a two‐way analysis of variance comparing relative abundances of compounds from the phenylpropanoid pathway between transgenic lines and control.

## 3 DISCUSSION

Although the presence of a gene encoding CM has been reported in transcriptomes of migratory PPNs before (Bauters et al., 2014; Haegeman et al., 2011), this is the first time it has been functionally characterized in a migratory nematode. Chorismate is the final product of the shikimate pathway, a pathway that has only been reported in plants and microorganisms, but not in animals (Herrmann, 1995). The first report of CM from a nematode was in *M. javanica* (Lambert et al., 1999), where it was thought to be involved in the establishment of a feeding site within the host. Indole‐3‐acetic acid (auxin, IAA) levels were reported to be lowered due to expression of this *CM* in plant roots, which had an influence on root development. This observation is in contradiction with the findings that IAA is important for feeding site initiation, while the nematode‐secreted CM was hypothesized to be a vital element in this process (Goverse et al., 2000).

The CM protein produced by *H. oryzae* contains a unique N‐terminal serine/histidine‐rich domain and a conserved catalytic domain with CM activity. Root‐knot nematodes lack an N‐terminal domain preceding the CM catalytic domain. Cyst nematode CMs do have an N‐terminal domain, but lack homology to the serine/histidine rich domain found in *H. oryzae*. Vanholme et al. (2009) proved that the N‐terminal sequence was not needed for CM activity in *Heterodera schachtii*, similar to the here‐obtained results for HoCM. More research is needed to elucidate the function of the N‐terminal region present in cyst nematodes and *H. oryzae*. Because it is not needed for enzymatic activity, it is possible that this region has another function, for example interaction with host proteins.

A geneencoding ICM was reported in the transcriptome of *H. oryzae* (Bauters et al., 2014), in *R. reniformis* (Wubben et al., 2010), and in the genome of the cyst nematode *G. rostochiensis* (Eves‐van den Akker et al., 2016). As Opperman et al. (2008) suggested while studying the genome of *M. hapla*, *ICM* has probably been acquired by horizontal gene transfer from bacteria. The sequence of *ICM* does not contain a predicted N‐terminal secretion signal, but the fact that the gene is conserved in other PPNs, while being absent in nematodes with a different lifestyle (Bauters et al., 2014), led us to believe it might be involved in plant parasitism. This hypothesis is strengthened by the fact that ICM is also present in the secretome and in the culture supernatants of phytopathogenic fungi, helping to increase susceptibility of the host (Soanes et al., 2008; El‐Bebany et al., 2010; Ismail et al., 2014). ICM is probably secreted by a nonclassical secretory pathway in fungi (Liu et al., 2014), but some fungal ICMs are predicted to have a secretion signal (Ismail et al., 2014). Further research is necessary to provide experimental evidence of ICM secretion by nematodes.

Although it is generally assumed that SA mediates resistance against biotrophic pathogens (Glazebrook, 2005) and *H. oryzae* kills the cells it is feeding from, in contrast to sedentary nematodes (De Vleesschauwer et al., 2013), SA was shown to be important for an adequate defence response against *H. oryzae* infection in rice (Nahar et al., 2012). On the other hand, the SA biosynthesis pathway appears changed after *H. oryzae* infection with up‐regulation of *OsPAL1* and down‐regulation of *OsICS* in shoots (Kyndt et al., 2012). SA biosynthesis in plants can occur through two different pathways, both starting from chorismate as a precursor (Vlot et al., 2009; reviewed in Lefevere et al., 2020). A secreted CM from *U. maydis* was able to reduce SA accumulation upon pathogen infection (Djamei et al., 2011). ICM, secreted by two fungal pathogens (*Verticillium dahliae* and *Phytophthora sojae*), reduces the SA content at least 3‐fold upon transient expression in *Nicotiana  benthamiana* leaves, rendering the plant more vulnerable to various pathogens (Liu et al., 2014). According to the results presented in this research, HoICM and HoCM do not have an effect on SA. However, upon interpreting these results, one has to keep in mind that the RNA‐Seq and metabolome data presented here came from plants that were not challenged with a pathogen, in contrast to the data of Liu et al. (2014) and Djamei et al. (2011). A GO enrichment analysis performed on RNA‐Seq data from *HoICM‐*, *HoCM_FULL‐*, and *HoCM_CAT*‐expressing rice lines, together with a metabolome analysis, points towards changes in phenylpropanoid content. The GO term “secondary metabolic process” and its child term “phenylpropanoid metabolic process” were reduced in the three different transgenic lines. Both ICM and CM act upon chorismate (or chorismate‐derived metabolites), which is the starting point of the phenylpropanoid pathway (Dempsey et al., 2011). These data were backed up by results extracted from the metabolome analysis on the transgenic lines. Looking at identified compounds, phenylpropanoid content was lower in plants expressing *HoCM*, whereas results for plants expressing *HoICM* were less clear. The fact that the *PAL* genes, considered as the starting point of the phenylpropanoid pathway, are often up‐ and down‐regulated in resistant and susceptible interactions, respectively, shows that phenylpropanoids are important defence molecules. This was shown in several plant–pathogen interaction experiments (Gao et al., 2008; Uehara et al., 2010; Feng et al., 2011; Khanam et al., 2018). In contrast to our observations, Djamei and colleagues (2011) reported an increase in phenylpropanoid biosynthesis products in plants infected with *U. maydis*, which indicates that chorismate is directed into the phenylpropanoid pathway. CM originating from *U. maydis* was also able to reduce the SA content of maize upon infection (Djamei et al., 2011). A recent study showed that transient expression of CM originating from the PPN *M. incognita* was able to reduce the SA content of tobacco leaves after *Phytophthora* infection (Wang et al., 2018). Our results showed that unchallenged rice plants expressing *HoCM* or *HoICM* did not change their SA content in roots or shoots. This observation was strengthened by the fact that no SA‐responsive genes (e.g., *OsWRKY45* or *OsNPR1*) were differentially regulated according to the transcriptome analysis.

Genes involved in “Diterpenoid phytoalexin metabolic process” were inhibited in *HoCM_FULL‐* and *HoCM_CAT*‐expressing plants. Diterpenoid phytoalexins (DPs) have several functions in plants. Their antimicrobial properties have been well documented. DPs accumulate in infection spots where necrotic tissue starts to develop (Huffaker et al., 2011). Transgenic lines deficient in *OsCPS4*, a phytoalexin biosynthetic gene, showed increased susceptibility to rice blast fungus (Toyomasu et al., 2014). Also in nematodes it was shown that induction of DP biosynthesis increases resistance against nematodes (Verbeek et al., 2019). Some terpenoids might be involved in nematode resistance by accumulation in lesions near the infection site (Veech, 1982), so a reduced terpenoid content could influence susceptibility to nematodes. The mechanism by which expression of *HoCM* or *HoICM* influences the diterpenoid phytoalexin biosynthesis pathway remains unclear. So far, it was only suggested that the phenylpropanoid and terpenoid pathway compete for carbon and that carbon flow into these two pathways is tightly regulated (Xie et al., 2008).

The data generated in this paper suggest that the immune system of the host is altered by lowering secondary metabolite content upon secretion of CM and ICM by *H. oryzae*. Further research is needed to pinpoint the exact metabolites responsible for the drop in immunity and the mechanism by which HoCM and HoICM are able to do this.

## 4 EXPERIMENTAL PROCEDURES

### 4.1 Nematode DNA extraction and gene amplification

Genomic DNA was extracted from a batch of mixed stages of *H. oryzae* or a single hand‐picked nematode as previously described by Bolla et al. (1988), with some minor modifications. Briefly, nematodes were sonicated three times for 10 s to break cell membranes. Samples were incubated for 1 hr at 65°C after which DNA was isolated using phenol and chloroform. Isolated DNA and a cDNA library (Bauters et al., 2014) were used to amplify *HoCM* and *HoICM* by PCR with gene‐specific primers (see Table S6). PCR fragments were cloned into pGEM‐T (Promega) according to the standard thymine/adenine protocol and sequenced by the Sanger method (LGC) to verify the insert.

### 4.2 Bioinformatic analysis

Several blast programs (blastn and blastx), available on the NCBI server (http://www.ncbi.nlm.nih.gov/), were used for sequence similarity searches in EST and nonredundant protein databases. Alignments were generated with BioEdit (Hall, 1999). Putative N‐terminal secretion signals were detected by SignalP 4.1 (Petersen et al., 2011). Protein domains were identified by searching the Pfam 27.0 database (Punta et al., 2011). Protein 3D models were constructed with the Protein Homology/anologY Recognition Engine v. 2.0 (Phyre2) (Punta et al., 2011) and visualized by PyMOL 1.6 (http://www.pymol.org/).

### 4.3 Activity assays

Two *HoCM* fragments (HoCMFull and HoCMCat) were amplified by PCR using the full cDNA sequence as template (primer sequences can be found in Table S6). The first fragment comprised the full sequence of CM without sequence encoding the signal peptide, the second fragment only contained the sequence encoding the catalytic domain (base pair 258 to 747). The complementation assay was performed as described by Vanholme et al. (2009).

HoICM activity was tested using *entB*‐mutated AN192 *E. coli* cells. AN192 cells are unable to grow in iron‐limiting conditions. *HoICM* was amplified by a two‐step PCR starting from cDNA as template to add attB sites to the fragment (primer sequences can be found in Table S6). The fragment was cloned into the destination vector pDEST17 using the standard Gateway technology protocol (Life Technologies). The pDEST17 vector carrying *HoICM* was transformed via heat shock into *E. coli* AN192 cells. *E. coli* AN192 cells transformed with the empty vector were used as negative control. These cells were grown overnight at 37°C in Luria‐Bertani (LB) medium with 100 mg/L carbenicillin. Optical density (OD) was measured at 600 nm and equal amounts of bacteria were added to fresh medium containing different concentrations of 2,2ʹ‐bipyridyl to create iron‐limiting conditions (0, 100, 150, and 200 µM). These different types of media were inoculated with *E. coli* AN192 cells carrying *HoICM* or the empty vector. OD was measured at 600 nm at 15, 24, and 48 hr postinoculation. Results were statistically analysed with a Mann–Whitney test. The experiment was performed twice.

### 4.4 Plant transformation

cDNA sequences of *HoICM* and *HoCM* were amplified by PCR on a cDNA library (Bauters et al., 2014). Primer sequences are provided in Table S6. Expression vectors (pMBb7Fm21GW‐UBIL, obtained from the Center for Plant Systems Biology [VIB, Belgium]) were created for three different constructs: *HoICM* (containing the complete open reading frame), *HoCM* without secretion signal (HoCM_FULL), and only the catalytic domain of *HoCM* (*HoCM_CAT*). Vectors were created by standard Gateway cloning. Cloned sequences as well as position in the vector were verified by Sanger sequencing. Rice transformation was done as described by Ji et al. (2015). Leaf samples were taken and DNA was extracted to confirm the presence of the gene of interest by PCR using gene‐specific primers (Table S6). DNA was extracted by heating a leaf sample at 95°C for 15 min in 400 µl of extraction buffer (200 mM Tris.HCl, 250 mM NaCl, 25 mM ethylenediaminetetraacetic acid [EDTA] and 0.5% sodium dodecyl sulphate [SDS]). Afterwards 50 µl chloroform was added. The sample was centrifuged and 200 µl of isopropanol was added to 200 µl of the upper aqueous phase to precipitate the DNA.

### 4.5 RNAextraction, sequencing, and validation

Rice plants were grown under greenhouse conditions at 30°C. In the flowering stage, leaf samples were taken from three plants of a line transformed with the empty vector, and three plants of a line expressing *HoCM_FULL* or *HoCM_CAT*. Due to limited seed availability, the line for which most seeds per transformant were available was chosen. Because of technical reasons only two plants were used for lines expressing *HoICM*. RNA was extracted using the Nucleospin RNA extraction kit (Machery‐Nagel) according to the manufacturer's guidelines with an additional DNase I (Thermo Scientific) treatment. At least 4 µg of RNA of each sample was sent for RNA sequencing (Microsynth). An mRNA library was constructed using the Illumina TruSeq RNA sample preparation kit. The library was sequenced using a NextSeq platform (Illumina). To validate RNA‐Seq results, a set of genes was selected randomly to perform RT‐qPCR. RNA was extracted with the RNeasy Plant Mini kit (Qiagen). cDNA was synthesized and a RT‐qPCR was performed with *EIF5c* and *EXP NARCAI* as reference genes to normalize expression results. Primers can be found in Table S6.

### 4.6 Mapping and gene expression profiling

Reads from all libraries were mapped to the *O. sativa* ssp. *japonica* reference genome (IRGSP‐1.0) using the Spliced Transcripts Alignment to a Reference (STAR, v2.3.1) software with standard settings (Dobin et al., 2013). The GenomicFeatures (relying on Ensemble's BioMart osativa_eg_gene dataset) and GenomicAlignment R/BioConductor packages were used to obtain an overview of all exons per gene (makeTranscriptDbFromBiomart and exonsBy functions), respectively, to quantify expression per sample for these genes starting from the STAR output (readGAlignmentsFromBam and countOverlaps functions). Additional gene annotation information was obtained from Ensembl (BioMart, canonical identifiers only). Differential gene expression was assessed using the R‐package “EdgeR” version 3.8.5 (Robinson et al., 2010). Data were analysed using the generalized linear model (GLM) approach. Data were normalized using normalization factors calculated by the Trimmed Median of M‐values method with standard settings as implemented in the EdgeR package (Robinson and Oshlack, 2010). The proposed GLM was fitted to the design matrix using the Cox‐Reid profile‐adjusted likelihood method (McCarthy et al., 2012). Differential gene expression levels (Log_2_ fold change) were calculated and Benjamini and Hochberg adjusted (Benjamini and Hochberg, 1995) false discovery rates (FDR) were estimated to detect DEGs (FDR < 0.05). GO enrichment analysis was performed using agriGO (Du et al., 2010). Gene identifiers with their corresponding Log_2_FC were used as input in a Parametric Analysis of Gene Set Enrichment (PAGE) (Kim and Volsky, 2005). The Hochberg multitest adjustment method was performed (*p* < .05).

### 4.7 Phenolic profiling

Roots (approximately 5 mg of dry weight) were homogenized in liquid nitrogen and extracted with 1 ml of methanol. The methanol extract was then evaporated and the pellet dissolved in 200 μl of water/cyclohexane (1:1, vol/vol). Then, 10 μl of the aqueous phase was analysed via reverse‐phase ultrahigh performance liquid chromatography using an Acquity UPLC BEH C18 column. In addition to full MS analysis, a pooled sample was subjected to data‐dependent MS/MS analysis (exclusion duration = 10 s). Integration and alignment of the *m*/*z* features were performed via Progenesis QI software v. 2.1 (Waters Corporation). The normalization was set on “external standards” and was based on the dry weight of the samples.

For statistical analysis, the data were split into two sets. A first set comprised the two independent *HoICM* expression lines (HoICM1 and HoICM4) and the corresponding control line (*HoICM* set). In the second set, one *HoCM_FULL* expression line, two independent *HoCM_CAT* expression lines (HoCM_CAT2 and HoCM_CAT3), and the corresponding control line were included (*HoCM* set). Both sets were subjected to a one‐way ANOVA using the *lm()* function in R v. 3.4.2 (R_Core_Team, 2017) with a weighting factor included for the first set. For post hoc testing, the *pairwise.t.test(p.adjust* = *”bonferroni”)* function was applied. The false discovery rate (α = .01) was computed on the ANOVA model significance using the *p.adjust(method* = *”fdr”)* function. Additional filtering on the prerequisites of (a) a significant post hoc test for each of the transgenic lines and (b) abundance changes in the same direction for all transgenic lines as compared to the control lines yielded 54 and 17 differential *m*/*z* features (corresponding to 53 and 17 *m*/*z* feature groups) for the *HoICM* and *HoCM* sets, respectively.

Structural annotation was performed using a retention time window of 1 min, and both precursor ion and MS/MS identity searches. The precursor ion search (10 ppm tolerance) was based on a compound database constructed via instant JChem (ChemAxon), whereas MS/MS identities were obtained by matching against an in‐house mass spectral database (200 ppm fragment tolerance). As none of the MS/MS spectra of the differential *m*/*z* features could be identified via database matching, MS/MS spectral elucidation was attempted (see File S2) using gas phase fragmentation rules and in silico MS/MS elucidation software, that is, CSI:FingerID (Böcker and Dührkop, 2016; Dührkop et al., 2015) and CFM‐ID (Allen et al., 2015). A more detailed description of the phenolic profiling protocol can be found in File S2.

### 4.8 Nematode infection assay

The *M. graminicola* culture, originally isolated in the Philippines, was maintained on *O. sativa* "Nipponbare" in potting soil at 27°C (16/8 hr light regime). *H. oryzae* was obtained from infected rice roots sampled from fields in Myanmar. Nematodes were extracted from rice roots using a modified Baerman funnel technique. Rice seeds were first germinated on wet tissue paper at 27°C in the dark for 3 days. Germinated seedlings were transferred to PVC tubes containing sand and absorbent polymer substrate (Reversat et al., 1999). Two‐week old plants (T_1_ or T_2_ generation) were infected with 200 (*M. graminicola*) or 300 (*H. oryzae*) nematodes/plant. Two weeks after infection, nematode susceptibility was assessed by counting the number of galls (*M. graminicola*) or nematodes (*H. oryzae*) per root. Galls/nematodes were visualized by boiling roots in 0.8% acetic acid and 0.013% acid fuchsin for 3 min, after which they were washed under running tap water and destained in acid glycerol. Infection assays with *H. oryzae* were only conducted on a single line per construct due to difficulties in culturing *H. oryzae* under laboratory conditions.

## Supporting information


**FIGURE S1** Validation of RNA‐Seq results. Expression values were calculated compared to expression in the empty vector control (expression level set at 1). Normalization was done with two reference genes (EIF5C and EXP NARCAI). Expression values are plotted on a Log2‐scale. Expression values are a mean of two biological replicates (calculated by taking the average of three technical replicates). Statistical analysis was performed with REST 2009 software (asterisk indicates a significant difference, *p* < .05). Light grey bars indicate genes for which expression could not be detected in the respective overexpression line, expression values of these genes are equal to zero. Uridine diphosphate (UDP)‐glyctrsfr, UDP‐glycosyltransferase; GlutS, glutathione *S*‐transferase; ser/thr_kin, serine/threonine‐protein kinase; hist, histone H2B.3; LRR, LRR receptor‐like serine/threonine‐protein kinase; R‐like PK, receptor‐like protein kinase; α‐galac, α‐1,2‐galactosyltransferase; RIK, protein RIK; PM_ATPase, plasma membrane ATPase; gluc6, glucose‐6‐phosphate/phosphate translocator 2. In between brackets the log2FC according to RNA‐Seq is given. Bars represent standard errors, calculated with REST2009 software using Taylor’s series

**FIGURE S2** Root (A) and shoot (B) tissue of 3‐week‐old plants was harvested to quantify SA content on a ultra high performance liquid chromatography (U‐HPLC) system. Plant material was homogenized by grinding in liquid nitrogen (three individual plants per line were used). Extraction of 100 mg of plant material was performed using the modified Bieleski solvent after which it was filtrated and evaporated. Chromatographic separation was performed on a U‐HPLC system equipped with a Nucleodur C18 column (50 × 2 mm, 1.8 μm dp), using a mobile phase gradient consisting of acidified methanol and water. Mass spectrometric analysis was carried out in selected‐ion monitoring (SIM) mode with a Q Exactive™ Orbitrap mass spectrometer (Thermo Scientific), operating in both positive and negative electrospray ionization mode at a resolution of 70,000 full‐width at half maximum. Data were analysed with a nonparametric pairwise Kruskal–Wallis test. No significant differences were detected among the different lines. Ctrl, empty vector line

**FIGURE S3** Representation of the fragmentation pathways of the compound eluting at 3.26 min (id: 3.26_265.0921m/z)

**FIGURE S4** Representation of the fragmentation pathways of the compound eluting at 4.33 min (id: 4.33_309.1185m/z)

**TABLE S1** Table showing the Ct values for two control genes (Exp Narcai and EIF5C) and the transgene (A, HoCM; B, HoICM) that is expressed in the different transgenic lines and controls (wild‐type Nipponbare and empty vector line). Values that are underlined indicate that there was no expression for that gene. NA, not available

**TABLE S2** Overview of the output of RNA‐Seq on the different transgenic lines

**TABLE S3** Differential compounds in the transgenic lines overexpressing HoICM. FC, fold change; FDR, false discovery rate

**TABLE S4** Differential compounds in the transgenic lines overexpressing HoCM. FC, fold change; FDR, false discovery rate

**TABLE S5** MS/MS spectral information for a selection of compounds which are differentially abundant in HoCM or HoICM expressing lines. ID, compound identifier; tR, retention time; *m*/*z*, mass to charge ratio; Δppm, deviation of measured mass; MS/MS, relative abundances to the base peak of the MS/MS product ions are indicated between parentheses

**TABLE S6** Table with primer sequences used in this research

**FILE S1** List of differentially expressed genes in transgenic lines compared to the control according to the RNA‐Seq data

**FILE S2** Detailed protocol describing the metabolomics experiment

## Data Availability

Sequence data are available in GenBank at www.ncbi.nlm.nih.gov with accession numbers HoCM: KP297892, and HoICM: KP297893. The data that support the findings of this study are available from the corresponding author upon reasonable request.

## References

[mpp13003-bib-0001] Allen, F. , Greiner, R. and Wishart, D. (2015) Competitive fragmentation modeling of ESI‐MS/MS spectra for putative metabolite identification. Metabolomics, 11, 98–110.

[mpp13003-bib-0002] Babatola, J. (1981) Effect of pH, oxygen and temperature on the activity and survival of Hirschmanniella spp. Nematologica, 27, 285–291.

[mpp13003-bib-0003] Bauters, L. , Haegeman, A. , Kyndt, T. and Gheysen, G. (2014) Analysis of the transcriptome of *Hirschmanniella oryzae* to explore potential survival strategies and host–nematode interactions. Molecular Plant Pathology, 15, 352–363. 24279397 10.1111/mpp.12098 PMC6638887

[mpp13003-bib-0004] Benjamini, Y. and Hochberg, Y. (1995) Controlling the false discovery rate: a practical and powerful approach to multiple testing. Journal of the Royal Statistical Society. Series B (Methodological), 57, 289–300.

[mpp13003-bib-0005] Böcker, S. and Dührkop, K. (2016) Fragmentation trees reloaded. Journal of Cheminformatics, 8, 1–5. 26839597 10.1186/s13321-016-0116-8 PMC4736045

[mpp13003-bib-0006] Bolla, R. , Weaver, C. and Winter, R. (1988) Genomic differences among pathotypes of *Bursaphelenchus xylophilus* . Journal of Nematology, 20, 309–316. 19290214 PMC2618813

[mpp13003-bib-0007] Chook, Y.M. , Ke, H. and Lipscomb, W.N. (1993) Crystal structures of the monofunctional chorismate mutase from *Bacillus subtilis* and its complex with a transition state analog. Proceedings of the National Academy of Sciences of the United States of America, 90, 8600–8603. 8378335 10.1073/pnas.90.18.8600 PMC47405

[mpp13003-bib-0008] Davis, E.L. , Haegeman, A. and Kikuchi, T. (2011) Degradation of the plant cell wall by nematodes In: Jones J.T., Gheysen G. and Fenoll C. (Eds.) Genomics and Molecular Genetics of Plant‐Nematode Interactions. Dordrecht: Springer, pp. 255–272.

[mpp13003-bib-0009] De Vleesschauwer, D. , Gheysen, G. and Höfte, M. (2013) Hormone defense networking in rice: tales from a different world. Trends in Plant Science, 18, 555–565. 23910453 10.1016/j.tplants.2013.07.002

[mpp13003-bib-0010] Dempsey, D.M.A. , Vlot, A.C. , Wildermuth, M.C. and Klessig, D.F. (2011) Salicylic acid biosynthesis and metabolism. The Arabidopsis Book/American Society of Plant Biologists, 9, 1–24. 10.1199/tab.0156 PMC3268552 22303280

[mpp13003-bib-0011] Djamei, A. , Schipper, K. , Rabe, F. , Ghosh, A. , Vincon, V. , Kahnt, J. et al. (2011) Metabolic priming by a secreted fungal effector. Nature, 478, 395–400. 21976020 10.1038/nature10454

[mpp13003-bib-0012] Dobin, A. , Davis, C.A. , Schlesinger, F. , Drenkow, J. , Zaleski, C. , Jha, S. et al. (2013) STAR: ultrafast universal RNA‐seq aligner. Bioinformatics, 29, 15–21. 23104886 10.1093/bioinformatics/bts635 PMC3530905

[mpp13003-bib-0013] Doyle, E.A. and Lambert, K.N. (2003) *Meloidogyne javanica* chorismate mutase 1 alters plant cell development. Molecular Plant‐Microbe Interactions, 16, 123–131. 12575746 10.1094/MPMI.2003.16.2.123

[mpp13003-bib-0014] Du, Z. , Zhou, X. , Ling, Y. , Zhang, Z. and Su, Z. (2010) agriGO: a GO analysis toolkit for the agricultural community. Nucleic Acids Research, 38(suppl_2), W64–W70. 20435677 10.1093/nar/gkq310 PMC2896167

[mpp13003-bib-0015] Dührkop, K. , Shen, H. , Meusel, M. , Rousu, J. and Böcker, S. (2015) Searching molecular structure databases with tandem mass spectra using CSI: FingerID. Proceedings of the National Academy of Sciences of the United States of America, 112, 12580–12585. 26392543 10.1073/pnas.1509788112 PMC4611636

[mpp13003-bib-0016] El‐Bebany, A.F. , Rampitsch, C. and Daayf, F. (2010) Proteomic analysis of the phytopathogenic soilborne fungus *Verticillium dahliae* reveals differential protein expression in isolates that differ in aggressiveness. Proteomics, 10, 289–303. 20017145 10.1002/pmic.200900426

[mpp13003-bib-0017] Eves‐van den Akker, S. , Laetsch, D.R. , Thorpe, P. , Lilley, C.J. , Danchin, E.G.J. , Da Rocha, M. , et al. (2016) The genome of the yellow potato cyst nematode, Globodera rostochiensis, reveals insights into the basis of parasitism and virulence. Genome Biology, 17, 124. 27286965 10.1186/s13059-016-0985-1 PMC4901422

[mpp13003-bib-0018] Feng, J.‐X. , Cao, L. , Li, J. , Duan, C.‐J. , Luo, X.‐M. , Le, N. et al. (2011) Involvement of OsNPR1/NH1 in rice basal resistance to blast fungus *Magnaporthe oryzae* . European Journal of Plant Pathology, 131, 221–235.

[mpp13003-bib-0019] Gao, X. , Starr, J. , Göbel, C. , Engelberth, J. , Feussner, I. , Tumlinson, J. et al. (2008) Maize 9‐lipoxygenase ZmLOX3 controls development, root‐specific expression of defense genes, and resistance to root‐knot nematodes. Molecular Plant‐Microbe Interactions, 21, 98–109. 18052887 10.1094/MPMI-21-1-0098

[mpp13003-bib-0020] Glazebrook, J. (2005) Contrasting mechanisms of defense against biotrophic and necrotrophic pathogens. Annual Review of Phytopathology, 43, 205–227. 10.1146/annurev.phyto.43.040204.135923 16078883

[mpp13003-bib-0021] Goral, A.M. , Tkaczuk, K.L. , Chruszcz, M. , Kagan, O. , Savchenko, A. and Minor, W. (2012) Crystal structure of a putative isochorismatase hydrolase from *Oleispira antarctica* . Journal of Structural and Functional Genomics, 13, 27–36. 22350524 10.1007/s10969-012-9127-5 PMC3328404

[mpp13003-bib-0022] Goverse, A. , Overmars, H. , Engelbertink, J. , Schots, A. , Bakker, J. and Helder, J. (2000) Both induction and morphogenesis of cyst nematode feeding cells are mediated by auxin. Molecular Plant‐Microbe Interactions, 13, 1121–1129. 11043473 10.1094/MPMI.2000.13.10.1121

[mpp13003-bib-0023] Haeck, A. , Van Langenhove, H. , Harinck, L. , Kyndt, T. , Gheysen, G. , Höfte, M. et al. (2018) Trace analysis of multi‐class phytohormones in *Oryza sativa* using different scan modes in high‐resolution Orbitrap mass spectrometry: method validation, concentration levels, and screening in multiple accessions. Analytical and Bioanalytical Chemistry, 410, 4527–4539. 29796899 10.1007/s00216-018-1112-9

[mpp13003-bib-0024] Haegeman, A. , Joseph, S. and Gheysen, G. (2011) Analysis of the transcriptome of the root lesion nematode *Pratylenchus coffeae* generated by 454 sequencing technology. Molecular and Biochemical Parasitology, 178, 7–14. 21513748 10.1016/j.molbiopara.2011.04.001

[mpp13003-bib-0025] Hall, T.A. (1999) BioEdit: A User‐Friendly Biological Sequence Alignment Editor and Analysis Program for Windows 95/98/NT. Nucleic Acids Symposium Series. London: Information Retrieval Ltd., c1979–c2000, 95–98.

[mpp13003-bib-0026] Herrmann, K.M. (1995) The shikimate pathway: early steps in the biosynthesis of aromatic compounds. The Plant Cell, 7, 907–919. 12242393 10.1105/tpc.7.7.907 PMC160886

[mpp13003-bib-0027] Hewezi, T. and Baum, T.J. (2013) Manipulation of plant cells by cyst and root‐knot nematode effectors. Molecular Plant‐Microbe Interactions, 26, 9–16. 22809272 10.1094/MPMI-05-12-0106-FI

[mpp13003-bib-0028] Huang, G. , Dong, R. , Allen, R. , Davis, E.L. , Baum, T.J. and Hussey, R.S. (2005) Two chorismate mutase genes from the root‐knot nematode *Meloidogyne incognita* . Molecular Plant Pathology, 6, 23–30. 20565635 10.1111/j.1364-3703.2004.00257.x

[mpp13003-bib-0029] Huffaker, A. , Kaplan, F. , Vaughan, M.M. , Dafoe, N.J. , Ni, X. , Rocca, J.R. et al. (2011) Novel acidic sesquiterpenoids constitute a dominant class of pathogen‐induced phytoalexins in maize. Plant Physiology, 156, 2082–2097. 21690302 10.1104/pp.111.179457 PMC3149930

[mpp13003-bib-0030] Ismail, I. , Godfrey, D. and Able, A. (2014) Fungal growth, proteinaceous toxins and virulence of *Pyrenophora teres* f. *teres* on barley. Australasian Plant Pathology, 43, 535–546.

[mpp13003-bib-0031] Jaouannet, M. , Magliano, M. , Arguel, M.J. , Gourgues, M. , Evangelisti, E. , Abad, P. et al. (2013) The root‐knot nematode calreticulin Mi‐CRT is a key effector in plant defense suppression. Molecular Plant‐Microbe Interactions, 26, 97–105. 22857385 10.1094/MPMI-05-12-0130-R

[mpp13003-bib-0032] Ji, H. , Gheysen, G. , Ullah, C. , Verbeek, R. , Shang, C. , De Vleesschauwer, D. et al. (2015) The role of thionins in rice defence against root pathogens. Molecular Plant Pathology, 16, 870–881. 25676661 10.1111/mpp.12246 PMC6638518

[mpp13003-bib-0033] Jones, J.T. , Furlanetto, C. , Bakker, E. , Banks, B. , Blok, V. , Chen, Q. et al. (2003) Characterization of a chorismate mutase from the potato cyst nematode *Globodera pallida* . Molecular Plant Pathology, 4, 43–50. 20569361 10.1046/j.1364-3703.2003.00140.x

[mpp13003-bib-0034] Jones, J.T. , Furlanetto, C. and Phillips, M.S. (2007) The role of flavonoids produced in response to cyst nematode infection of *Arabidopsis thaliana* . Nematology, 9(), 671–677.

[mpp13003-bib-0035] Karakas, M. (2004) Life cycle and mating behavior of *Hirschmanniella oryzae* (nematoda: Pratylenchidae) on excised *Oryzae sativa* roots. Fen Bilimleri Dergisi, 25, 1–6.

[mpp13003-bib-0036] Khanam, S. , Bauters, L. , Singh, R.R. , Verbeek, R. , Haeck, A. , Sultan, S.M. et al. (2018) Mechanisms of resistance in the rice cultivar Manikpukha to the rice stem nematode *Ditylenchus angustus* . Molecular Plant Pathology, 19, 1391–1402. 28990717 10.1111/mpp.12622 PMC6638125

[mpp13003-bib-0037] Kim, S.‐Y. and Volsky, D.J. (2005) PAGE: parametric analysis of gene set enrichment. BMC Bioinformatics, 6, 144. 15941488 10.1186/1471-2105-6-144 PMC1183189

[mpp13003-bib-0038] Kyndt, T. , Nahar, K. , Haegeman, A. , De Vleesschauwer, D. , Höfte, M. and Gheysen, G. (2012) Comparing systemic defence‐related gene expression changes upon migratory and sedentary nematode attack in rice. Plant Biology, 14, 73–82. 22188265 10.1111/j.1438-8677.2011.00524.x

[mpp13003-bib-0039] Lambert, K.N. , Allen, K.D. and Sussex, I.M. (1999) Cloning and characterization of an esophageal‐gland‐specific chorismate mutase from the phytoparasitic nematode *Meloidogyne javanica* . Molecular Plant‐Microbe Interactions, 12, 328–336. 10188271 10.1094/MPMI.1999.12.4.328

[mpp13003-bib-0040] Lee, A.Y. , Karplus, P.A. , Ganem, B. and Clardy, J. (1995) Atomic structure of the buried catalytic pocket of *Escherichia coli* chorismate mutase. Journal of the American Chemical Society, 117, 3627–3628.

[mpp13003-bib-0041] Lefevere, H. , Bauters, L. and Gheysen, G. (2020) Salicylic acid biosynthesis in plants. Frontiers in Plant Science, 11, 338. 32362901 10.3389/fpls.2020.00338 PMC7182001

[mpp13003-bib-0042] Liu, T. , Song, T. , Zhang, X, , Yuan, H. , Su, L. , Li, W. , et al. (2014) Unconventionally secreted effectors of two filamentous pathogens target plant salicylate biosynthesis. Nature Communications, 5 (1), 4686. 10.1038/ncomms5686 PMC4348438 25156390

[mpp13003-bib-0043] McCarthy, D.J. , Chen, Y. and Smyth, G.K. (2012) Differential expression analysis of multifactor RNA‐Seq experiments with respect to biological variation. Nucleic Acids Research, 40, 4288–4297. 22287627 10.1093/nar/gks042 PMC3378882

[mpp13003-bib-0044] Nahar, K. , Kyndt, T. , Nzogela, Y.B. and Gheysen, G. (2012) Abscisic acid interacts antagonistically with classical defense pathways in rice–migratory nematode interaction. New Phytologist, 196, 901–913. 22985247 10.1111/j.1469-8137.2012.04310.x

[mpp13003-bib-0045] Nicol, P. , Gill, R. , Fosu‐Nyarko, J. and Jones, M.G. (2012) de novo analysis and functional classification of the transcriptome of the root lesion nematode, *Pratylenchus thornei*, after 454 GS FLX sequencing. International Journal for Parasitology, 42, 225–237. 22309969 10.1016/j.ijpara.2011.11.010

[mpp13003-bib-0046] Ökvist, M. , Dey, R. , Sasso, S. , Grahn, E. , Kast, P. and Krengel, U. (2006) 1.6 Å crystal structure of the secreted chorismate mutase from *Mycobacterium tuberculosis*: novel fold topology revealed. Journal of Molecular Biology, 357, 1483–1499. 16499927 10.1016/j.jmb.2006.01.069

[mpp13003-bib-0047] Opperman, C.H. , Bird, D.M. , Williamson, V.M. , Rokhsar, D.S. , Burke, M. , Cohn, J. et al. (2008) Sequence and genetic map of *Meloidogyne hapla*: a compact nematode genome for plant parasitism. Proceedings of the National Academy of Sciences of the United States of America, 105, 14802–14807. 18809916 10.1073/pnas.0805946105 PMC2547418

[mpp13003-bib-0048] Parsons, J.F. , Calabrese, K. , Eisenstein, E. and Ladner, J.E. (2003) Structure and mechanism of *Pseudomonas aeruginosa* PhzD, an isochorismatase from the phenazine biosynthetic pathway. Biochemistry, 42, 5684–5693. 12741825 10.1021/bi027385d

[mpp13003-bib-0049] Petersen, T.N. , Brunak, S. , von Heijne, G. and Nielsen, H. (2011) SignalP 4.0: discriminating signal peptides from transmembrane regions. Nature Methods, 8, 785–786. 21959131 10.1038/nmeth.1701

[mpp13003-bib-0050] Postma, W.J. , Slootweg, E.J. , Rehman, S. , Finkers‐Tomczak, A. , Tytgat, T.O. , van Gelderen, K. et al. (2012) The effector SPRYSEC‐19 of *Globodera rostochiensis* suppresses CC‐NB‐LRR‐mediated disease resistance in plants. Plant Physiology, 160, 944–954. 22904163 10.1104/pp.112.200188 PMC3461567

[mpp13003-bib-0051] Punta, M. , Coggill, P.C. , Eberhardt, R.Y. , Mistry, J. , Tate, J. , Boursnell, C. et al. (2011) The Pfam protein families database. Nucleic Acids Research, 40, D290–D301. 22127870 10.1093/nar/gkr1065 PMC3245129

[mpp13003-bib-0052] R_Core_Team (2017) R: A Language and Environment for Statistical Computing. Vienna, Austria: R Foundation for Statistical Computing [Online]. Available at: https://www.R‐project.org/. [Accessed 9 October 2020].

[mpp13003-bib-0054] Reversat, G. , Boyer, J. , Sannier, C. and Pando‐Bahuon, A. (1999) Use of a mixture of sand and water‐absorbent synthetic polymer as substrate for the xenic culturing of plant‐parasitic nematodes in the laboratory. Nematology, 1, 209–212.

[mpp13003-bib-0055] Robinson, M.D. , McCarthy, D.J. and Smyth, G.K. (2010) edgeR: a bioconductor package for differential expression analysis of digital gene expression data. Bioinformatics, 26, 139–140. 19910308 10.1093/bioinformatics/btp616 PMC2796818

[mpp13003-bib-0056] Robinson, M.D. and Oshlack, A. (2010) A scaling normalization method for differential expression analysis of RNA‐seq data. Genome Biology, 11, R25. 20196867 10.1186/gb-2010-11-3-r25 PMC2864565

[mpp13003-bib-0057] Soanes, D.M. , Alam, I. , Cornell, M. , Wong, H.M. , Hedeler, C. , Paton, N.W. et al. (2008) Comparative genome analysis of filamentous fungi reveals gene family expansions associated with fungal pathogenesis. PLoS One, 3, e2300. 18523684 10.1371/journal.pone.0002300 PMC2409186

[mpp13003-bib-0058] Toyomasu, T. , Usui, M. , Sugawara, C. , Otomo, K. , Hirose, Y. , Miyao, A. et al. (2014) Reverse‐genetic approach to verify physiological roles of rice phytoalexins: characterization of a knockdown mutant of *OsCPS4* phytoalexin biosynthetic gene in rice. Physiologia Plantarum, 150, 55–62. 23621683 10.1111/ppl.12066

[mpp13003-bib-0059] Uehara, T. , Sugiyama, S. , Matsuura, H. , Arie, T. and Masuta, C. (2010) Resistant and susceptible responses in tomato to cyst nematode are differentially regulated by salicylic acid. Plant and Cell Physiology, 51, 1524–1536. 20660227 10.1093/pcp/pcq109

[mpp13003-bib-0060] Vanholme, B. , Kast, P. , Haegeman, A. , Jacob, J. , Grunewald, W. and Gheysen, G. (2009) Structural and functional investigation of a secreted chorismate mutase from the plant‐parasitic nematode *Heterodera schachtii* in the context of related enzymes from diverse origins. Molecular Plant Pathology, 10, 189–200. 19236568 10.1111/j.1364-3703.2008.00521.x PMC6640496

[mpp13003-bib-0061] Veech, J.A. (1982) Phytoalexins and their role in the resistance of plants to nematodes. Journal of Nematology, 14, 2–9. 19295667 PMC2618140

[mpp13003-bib-0062] Verbeek, R.E.M. , Van Buyten, E. , Alam, M.Z. , De Vleesschauwer, D. , Van Bockhaven, J. , Asano, T. , et al. (2019) Jasmonate‐induced defense mechanisms in the belowground antagonistic interaction between *Pythium arrhenomanes* and *Meloidogyne graminicola* in Rice. Frontiers in Plant Science, 10, 1515. 31824540 10.3389/fpls.2019.01515 PMC6883413

[mpp13003-bib-0063] Vlot, A.C. , Dempsey, D.M.A. and Klessig, D.F. (2009) Salicylic acid, a multifaceted hormone to combat disease. Annual Review of Phytopathology, 47, 177–206. 10.1146/annurev.phyto.050908.135202 19400653

[mpp13003-bib-0064] Wang, X. , Xue, B. , Dai, J. , Qin, X. , Liu, L. , Chi, Y. et al. (2018) A novel *Meloidogyne incognita* chorismate mutase effector suppresses plant immunity by manipulating the salicylic acid pathway and functions mainly during the early stages of nematode parasitism. Plant Pathology, 67, 1436–1448.

[mpp13003-bib-0065] Wubben, M.J. , Callahan, F.E. and Scheffler, B.S. (2010) Transcript analysis of parasitic females of the sedentary semi‐endoparasitic nematode *Rotylenchulus reniformis* . Molecular and Biochemical Parasitology, 172, 31–40. 20346373 10.1016/j.molbiopara.2010.03.011

[mpp13003-bib-0066] Xie, Z. , Kapteyn, J. and Gang, D.R. (2008) A systems biology investigation of the MEP/terpenoid and shikimate/phenylpropanoid pathways points to multiple levels of metabolic control in sweet basil glandular trichomes. The Plant Journal, 54, 349–361. 18248593 10.1111/j.1365-313X.2008.03429.x

