# Supplementary material for: Chorismate mutase and isochorismatase, two potential effectors of the migratory nematode Hirschmanniella oryzae, increase host susceptibility by manipulating secondary metabolite content of rice
Source: Mol Plant Pathol. 2020 Oct 20;21(12):1634–46. doi: 10.1111/mpp.13003 (PMC7694671; doi:10.1111/mpp.13003)
Supplement: Supplementary file 1 — FIGURE S1 Validation of RNA‐Seq results. Expression values were calculated compared to expression in the empty vector control (expression level set at 1). Normalization was done with two reference genes (EIF5C and EXP NARCAI). Expression values are plotted on a Log2‐scale. Expression values are a mean of two biological replicates (calculated by taking the average of three technical replicates). Statistical analysis was performed with REST 2009 software (asterisk indicates a significant difference, p < .05). Light grey bars indicate genes for which expression could not be detected in the respective overexpression line, expression values of these genes are equal to zero. Uridine diphosphate (UDP)‐glyctrsfr, UDP‐glycosyltransferase; GlutS, glutathione S‐transferase; ser/thr_kin, serine/threonine‐protein kinase; hist, histone H2B.3; LRR, LRR receptor‐like serine/threonine‐protein kinase; R‐like PK, receptor‐like protein kinase; α‐galac, α‐1,2‐galactosyltransferase; RIK, protein RIK; PM_ATPase, plasma membrane ATPase; gluc6, glucose‐6‐phosphate/phosphate translocator 2. In between brackets the log2FC according to RNA‐Seq is given. Bars represent standard errors, calculated with REST2009 software using Taylor’s series [file MPP-21-1634-s001.docx]

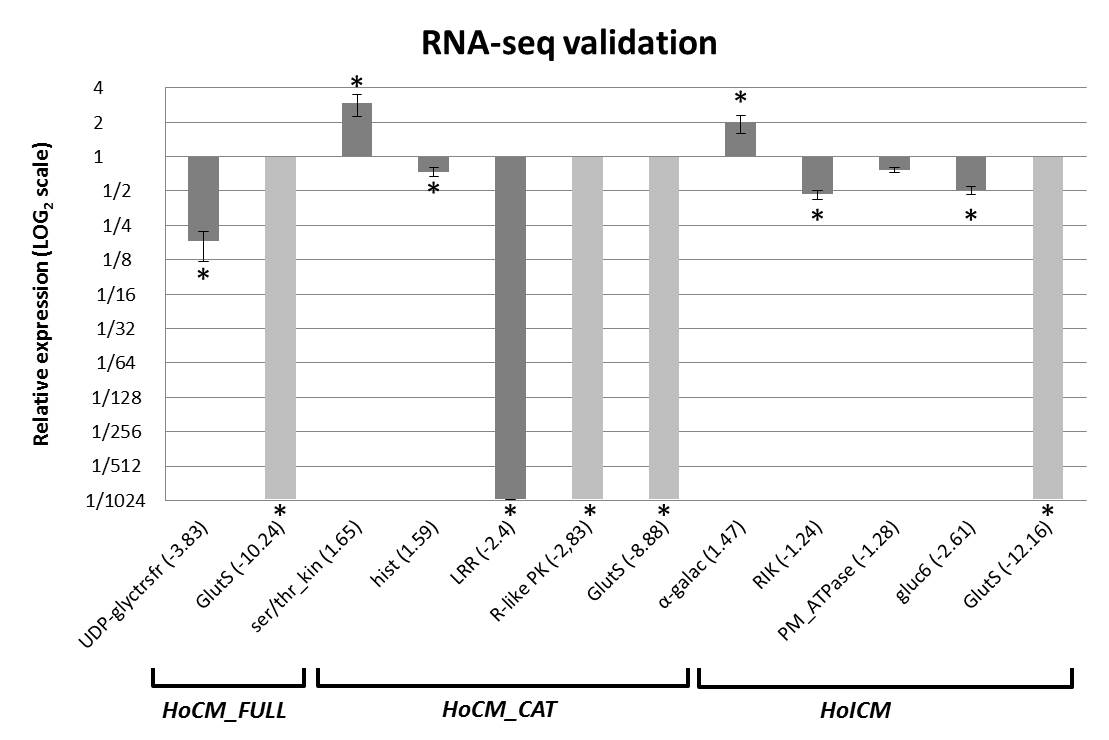


Supplementary figure S1: Validation of RNA-seq results. Expression values were calculated compared to expression in the empty vector control (expression level set at 1). Normalization was done with two reference genes (EIF5C and EXP NARCAI). Expression values are plotted on a Log_2_-scale. Expression values are a mean of two biological replicates (calculated by taking the average of three technical replicates). Statistical analysis was performed with REST 2009 software (an asterisk (*) indicates a significant difference, p<0.05). Light grey bars indicate genes of which expression could not be detected in the respective overexpression line, expression values of these genes are equal to zero. UDP-glyctrsfr: UDP-glycosyltransferase, GlutS: Glutathione S-transferase, ser/thr_kin: Serine/threonine-protein kinase, hist: Histone H2B.3, LRR: LRR receptor-like serine/threonine-protein kinase, R-like PK: Receptor-like protein kinase, α-galac: α-1,2-galactosyltransferase, RIK: Protein RIK, PM_ATPase: Plasma membrane ATPase, gluc6: Glucose-6-phosphate/phosphate translocator 2. In between brackets the log_2_FC according to RNA-sequencing is given. Bars represent standard errors, calculated with REST2009 software using Taylor’s series.
