# Supplementary material for: Chorismate mutase and isochorismatase, two potential effectors of the migratory nematode Hirschmanniella oryzae, increase host susceptibility by manipulating secondary metabolite content of rice
Source: Mol Plant Pathol. 2020 Oct 20;21(12):1634–46. doi: 10.1111/mpp.13003 (PMC7694671; doi:10.1111/mpp.13003)
Supplement: Supplementary file 2 — FIGURE S2 Root (A) and shoot (B) tissue of 3‐week‐old plants was harvested to quantify SA content on a ultra high performance liquid chromatography (U‐HPLC) system. Plant material was homogenized by grinding in liquid nitrogen (three individual plants per line were used). Extraction of 100 mg of plant material was performed using the modified Bieleski solvent after which it was filtrated and evaporated. Chromatographic separation was performed on a U‐HPLC system equipped with a Nucleodur C18 column (50 × 2 mm, 1.8 μm dp), using a mobile phase gradient consisting of acidified methanol and water. Mass spectrometric analysis was carried out in selected‐ion monitoring (SIM) mode with a Q Exactive™ Orbitrap mass spectrometer (Thermo Scientific), operating in both positive and negative electrospray ionization mode at a resolution of 70,000 full‐width at half maximum. Data were analysed with a nonparametric pairwise Kruskal–Wallis test. No significant differences were detected among the different lines. Ctrl, empty vector line [file MPP-21-1634-s002.docx]

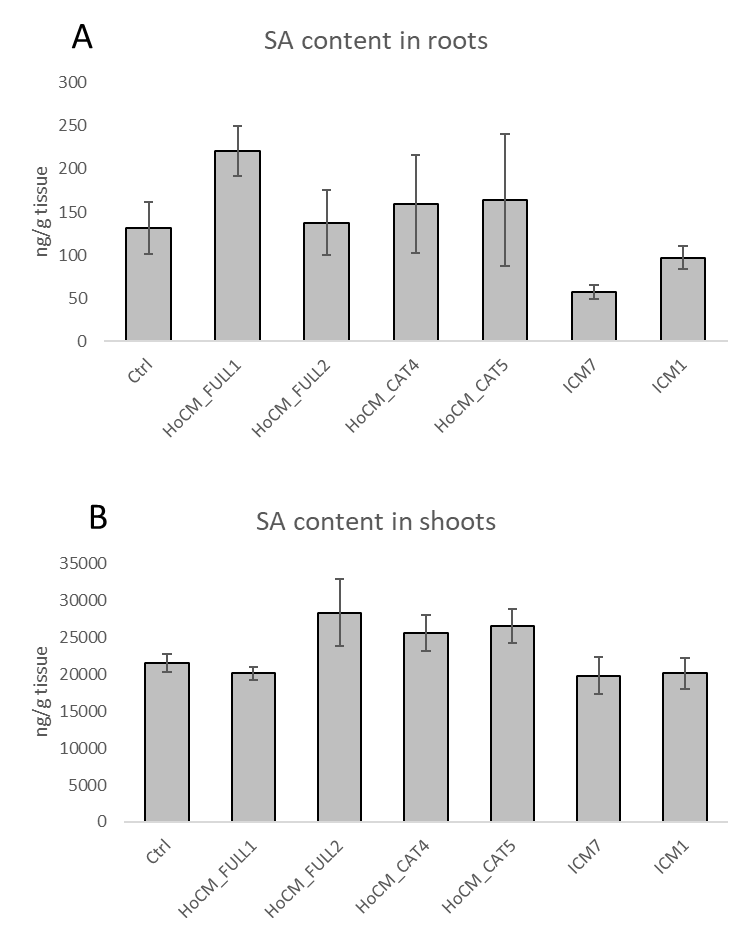


Supplementary figure S2: Root (A) and shoot (B) tissue of three week old plants was harvested to quantify SA-content on a U-HPLC system. Plant material was homogenized by grinding in liquid nitrogen (three individual plants per line were used). Extraction of 100mg plant material was performed using the modified Bieleski solvent after which it was filtrated and evaporated. Chromatographic separation was performed on a U-HPLC system equipped with a Nucleodur C18 column (50 x 2 mm; 1.8 μm dp), using a mobile phase gradient consisting of acidified methanol and water. Mass spectrometric analysis was carried out in selected-ion monitoring (SIM) mode with a Q Exactive™ Orbitrap mass spectrometer (Thermo Scientific), operating in both positive and negative electrospray ionization mode at a resolution of 70.000 full width at half maximum. Data were analyzed with a non-parametric pairwise Kruskal-Wallis test. No significant differences were detected among the different lines. Ctrl: empty vector line.
