# Supplementary material for: Chorismate mutase and isochorismatase, two potential effectors of the migratory nematode Hirschmanniella oryzae, increase host susceptibility by manipulating secondary metabolite content of rice
Source: Mol Plant Pathol. 2020 Oct 20;21(12):1634–46. doi: 10.1111/mpp.13003 (PMC7694671; doi:10.1111/mpp.13003)
Supplement: Supplementary file 3 — FIGURE S3 Representation of the fragmentation pathways of the compound eluting at 3.26 min (id: 3.26_265.0921m/z) [file MPP-21-1634-s003.docx]

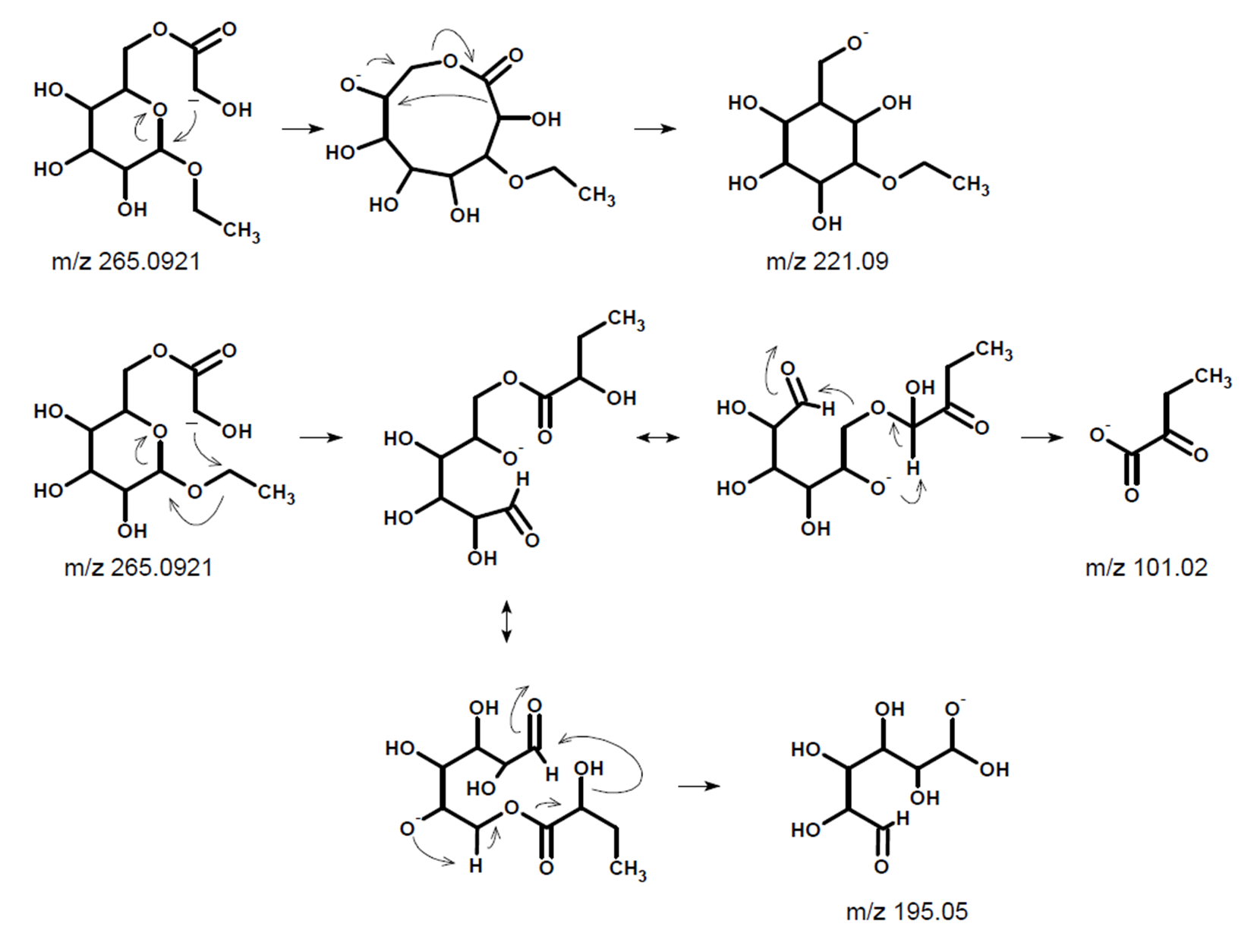


Supplementary figure S3:Representation of the fragmentation pathways of the compound eluting at 3.26 min. (id: 3.26_265.0921m/z).
