# Supplementary material for: Chorismate mutase and isochorismatase, two potential effectors of the migratory nematode Hirschmanniella oryzae, increase host susceptibility by manipulating secondary metabolite content of rice
Source: Mol Plant Pathol. 2020 Oct 20;21(12):1634–46. doi: 10.1111/mpp.13003 (PMC7694671; doi:10.1111/mpp.13003)
Supplement: Supplementary file 4 — FIGURE S4 Representation of the fragmentation pathways of the compound eluting at 4.33 min (id: 4.33_309.1185m/z) [file MPP-21-1634-s004.docx]

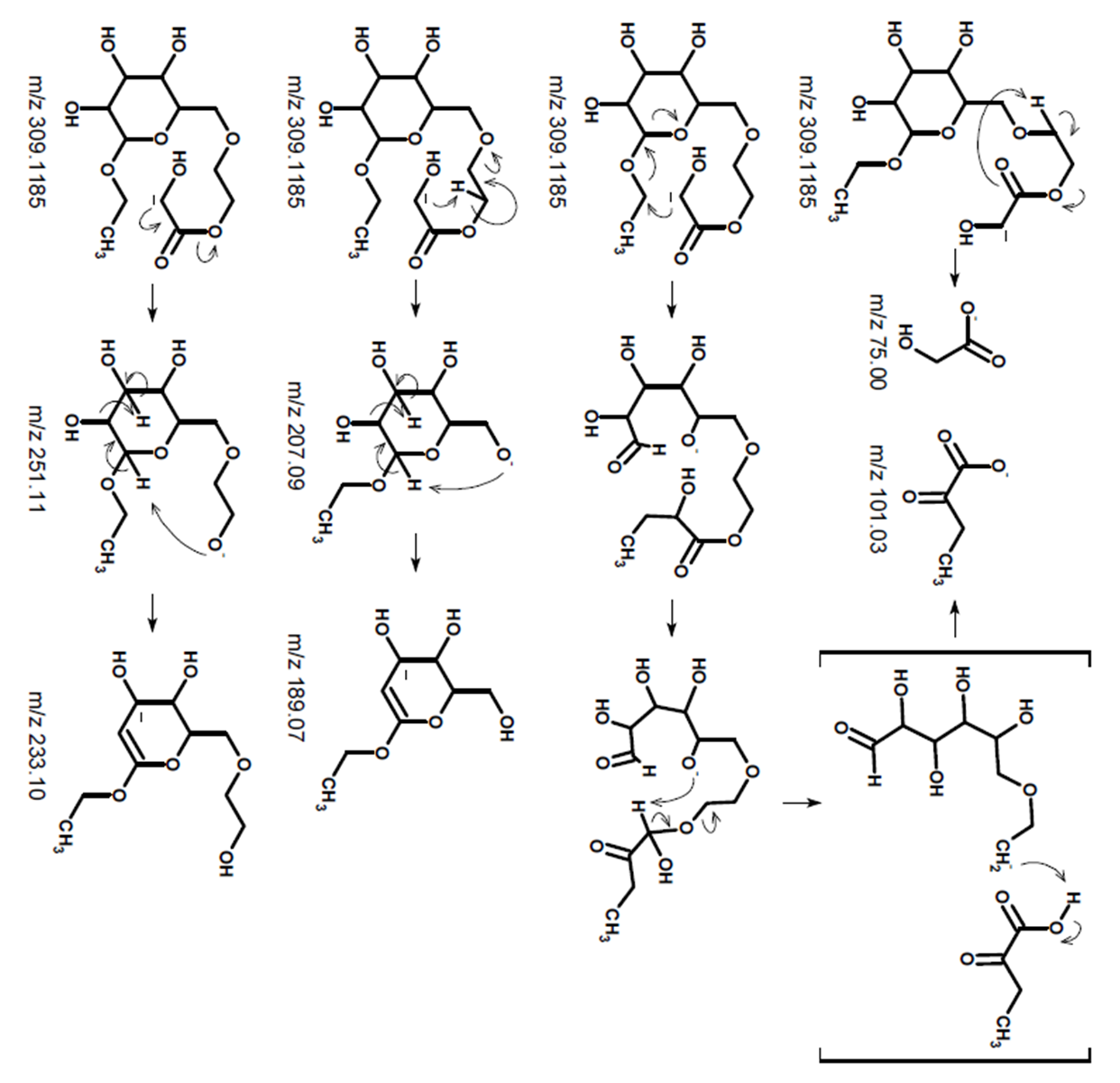


Supplementary figure S4: Representation of the fragmentation pathways of the compound eluting at 4.33 min. (id: 4.33_309.1185m/z).
