# Supplementary material for: Chorismate mutase and isochorismatase, two potential effectors of the migratory nematode Hirschmanniella oryzae, increase host susceptibility by manipulating secondary metabolite content of rice
Source: Mol Plant Pathol. 2020 Oct 20;21(12):1634–46. doi: 10.1111/mpp.13003 (PMC7694671; doi:10.1111/mpp.13003)
Supplement: Supplementary file 5 — TABLE S1 Table showing the Ct values for two control genes (Exp Narcai and EIF5C) and the transgene (A, HoCM; B, HoICM) that is expressed in the different transgenic lines and controls (wild‐type Nipponbare and empty vector line). Values that are underlined indicate that there was no expression for that gene. NA, not available [file MPP-21-1634-s005.docx]

Supplementary table S1: Table showing the Ct-values for two control genes (Exp Narcai and EIF5C) and the transgene (A: HoCM, B: HoICM) that is expressed in the different transgenic lines (primary transformants) and controls (Wild type Nipponbare and Empty vector line). Values that are underlined indicate that there was no expression for that gene. (NA: Not Available)

| **A Chorismate mutase** | | | |  | **B Isochorismatase** | | | |
| --- | --- | --- | --- | --- | --- | --- | --- | --- |
|  | **Exp Narcai** | **EIF5C** | **HoCM** |  |  | **Exp Narcai** | **EIF5C** | **HoICM** |
| **HoCM_CAT1** | 18.7 | 23.1 | 27.8 |  | **HoICM1** | 16.3 | 17.4 | 16.8 |
| **HoCM_CAT2** | 16.9 | 19.3 | 20.8 |  | **HoICM2** | NA | NA | NA |
| **HoCM_CAT3** | 25 | 29.6 | 27.7 |  | **HoICM3** | 16.4 | 17.3 | 31.5 |
| **HoCM_CAT4** | 17.7 | 20.7 | 21.5 |  | **HoICM4** | 16.8 | 17.9 | 17.0 |
| **HoCM_CAT5** | 24 | 28.1 | 27.2 |  | **HoICM5** | NA | NA | NA |
| **HoCM_FULL1** | 19.1 | 23.6 | 25.1 |  | **HoICM6** | NA | NA | NA |
| **HoCM_FULL2** | 17.2 | 20.2 | 22.9 |  | **HoICM7** | 16.7 | 17.6 | 16.8 |
| **Wild type** | 16.8 | 18.1 | 38.2 |  | **HoICM8** | 17.1 | 18 | 32.2 |
| **Empty vector** | 17 | 18.3 | 38.3 |  | **Wild type** | 17 | 18.8 | 32.9 |
|  |  |  |  |  | **Empty vector** | 16.1 | 17.7 | 35.6 |
