# Supplementary material for: Chorismate mutase and isochorismatase, two potential effectors of the migratory nematode Hirschmanniella oryzae, increase host susceptibility by manipulating secondary metabolite content of rice
Source: Mol Plant Pathol. 2020 Oct 20;21(12):1634–46. doi: 10.1111/mpp.13003 (PMC7694671; doi:10.1111/mpp.13003)
Supplement: Supplementary file 6 — TABLE S2 Overview of the output of RNA‐Seq on the different transgenic lines [file MPP-21-1634-s006.docx]

Supplementary table S2: Overview of the output of the RNA-sequencing on the different transgenic lines

| **Sample** |  | **Total number of** | **Uniquely mapped** | **Reads mapped to** | **Unmapped reads** |
| --- | --- | --- | --- | --- | --- |
|  |  | **sequence reads** | **reads** | **multiple loci** |  |
| **Empty vector** | rep.1 | 13,428,749 | 11,747,649 | 868,395 | 648,609 |
|  | rep.2 | 14,958,403 | 9,925,189 | 3,493,130 | 683,599 |
|  | rep.3 | 15,227,582 | 11,916,083 | 1,899,690 | 846,654 |
| ***HoCM_FULL2*** | rep.1 | 10,809,981 | 8,085,457 | 1,643,525 | 590,225 |
|  | rep.2 | 14,897,494 | 12,883,852 | 943,430 | 704,651 |
|  | rep.3 | 13,041,032 | 10,931,807 | 1,148,182 | 640,315 |
| ***HoCM_CAT4*** | rep.1 | 13,825,284 | 11,819,619 | 979,122 | 789,424 |
|  | rep.2 | 12,350,758 | 9,332,242 | 1,768,786 | 707,698 |
|  | rep.3 | 10,809,639 | 9,063,044 | 916,922 | 597,773 |
| ***HoICM7*** | rep.1 | 13,648,763 | 11,717,531 | 960,184 | 700,182 |
|  | rep.2 | 14,460,569 | 12,562,608 | 910,609 | 712,906 |
