# Supplementary material for: Chorismate mutase and isochorismatase, two potential effectors of the migratory nematode Hirschmanniella oryzae, increase host susceptibility by manipulating secondary metabolite content of rice
Source: Mol Plant Pathol. 2020 Oct 20;21(12):1634–46. doi: 10.1111/mpp.13003 (PMC7694671; doi:10.1111/mpp.13003)
Supplement: Supplementary file 7 — TABLE S3 Differential compounds in the transgenic lines overexpressing HoICM. FC, fold change; FDR, false discovery rate [file MPP-21-1634-s007.docx]

Supplementary table S3: Differential compounds in the transgenic lines overexpressing HoICM. FC: Fold change, FDR: False Discovery Rate.

| **ID** | **Trivial name** | **Mean abundance** | | | **FC** | **FDR** |
| --- | --- | --- | --- | --- | --- | --- |
|  |  | **CTRL** | **HoICM1** | **HoICM4** |  |  |
| 3.26_265.0921m/z | 1-O-ethyl-6-O-glycoloyl hexose | 6.09 | 190.04 | 27.12 | 17.84 | 0.002 |
| 11.36_341.1056m/z | Unknown | 0.02 | 0.27 | 0.25 | 11.37 | 0.006 |
| 4.30_286.0923m/z | Unknown | 1.01 | 7.94 | 12.65 | 10.22 | 0.003 |
| 4.33_309.1185m/z | (1-O-ethyl)hexosyl(6->)ethylene glycol glycolate | 16.55 | 249.29 | 54.78 | 9.19 | 0.007 |
| 5.34_228.0846m/z | Unknown | 0.08 | 0.92 | 0.37 | 8.02 | 0.002 |
| 5.07_487.1439m/z | Unknown | 4.3 | 13.67 | 16.54 | 3.51 | 0.009 |
| 4.93_409.1701m/z | Unknown | 18.05 | 52.31 | 49.83 | 2.83 | 0.007 |
| 11.67_360.2388m/z | Unknown | 156.9 | 53.82 | 90.7 | 0.46 | 0.008 |
| 13.19_360.2387m/z | Unknown | 66.66 | 20.03 | 38.96 | 0.44 | 0.005 |
| 14.38_243.1233m/z | Unknown | 21.04 | 7.79 | 10.66 | 0.44 | 0.01 |
| 12.98_360.2384m/z | Unknown | 51.61 | 13.54 | 30.32 | 0.42 | 0.002 |
| 12.66_316.2123m/z | Unknown | 40.68 | 10.7 | 23.68 | 0.42 | 0.004 |
| 12.28_316.2123m/z | Unknown | 40.06 | 9.2 | 22.72 | 0.4 | 0.001 |
| 10.76_314.1964m/z | Unknown | 28.04 | 6.91 | 15.34 | 0.4 | 0.003 |
| 10.35_244.1547m/z | Unknown | 9.78 | 1.66 | 5.55 | 0.37 | 0.001 |
| 15.91_342.2278m/z | Unknown | 25.81 | 5.5 | 13.4 | 0.37 | 0.004 |
| 16.28_342.2279m/z | Unknown | 65.71 | 12.94 | 35.13 | 0.37 | 0.002 |
| 2.90_461.1118m/z | Unknown | 118.06 | 60.69 | 25.54 | 0.37 | 0.003 |
| 17.02_344.2435m/z | Unknown | 21.35 | 4.39 | 10.6 | 0.35 | 0.004 |
| 12.38_358.2223m/z | Unknown | 4.01 | 0.7 | 1.99 | 0.34 | 0.005 |
| 11.11_249.0315m/z | Unknown | 37.46 | 8.33 | 16.59 | 0.33 | 0.006 |
| 12.39_413.1491m/z | Unknown | 15.5 | 2.32 | 7.96 | 0.33 | 0.001 |
| 17.15_344.2424m/z | Unknown | 12.51 | 1.86 | 6.37 | 0.33 | 0.002 |
| 11.98_360.2379m/z | Unknown | 12.09 | 1.49 | 6.34 | 0.32 | 0.001 |
| 12.40_316.2117m/z | Unknown | 9.61 | 1.09 | 5.09 | 0.32 | 0.001 |
| 15.65_345.1542m/z | Unknown | 3.64 | 0.74 | 1.6 | 0.32 | 0.002 |
| 14.09_270.1701m/z | Unknown | 5.64 | 0.89 | 2.69 | 0.32 | 0.004 |
| 10.99_314.1963m/z | Unknown | 9.34 | 1.43 | 4.33 | 0.31 | 0.004 |
| 2.95_181.9913m/z | Unknown | 10.24 | 1.86 | 4.41 | 0.31 | 0.005 |
| 11.81_358.2223m/z | Unknown | 8.98 | 1.03 | 4.2 | 0.29 | 0.003 |
| 12.71_314.1963m/z | Unknown | 9.55 | 1.28 | 4.27 | 0.29 | 0.001 |
| 11.77_314.1962m/z | Unknown | 1.88 | 0.08 | 0.91 | 0.26 | 0.001 |
| 16.78_342.2274m/z | Unknown | 22.87 | 2.13 | 9.88 | 0.26 | 0.002 |
| 15.87_272.1860m/z | Unknown | 5.61 | 0.64 | 2.27 | 0.26 | 0.002 |
| 21.99_342.2266m/z | Unknown | 2.61 | 0.15 | 1.11 | 0.24 | 0.001 |
| 12.04_314.1959m/z | Unknown | 1.41 | 0.13 | 0.54 | 0.24 | 0.003 |
| 21.01_342.2272m/z | Unknown | 10.06 | 1.23 | 3.53 | 0.24 | 0.004 |
| 1.75_262.0383m/z | Unknown | 26.39 | 8.6 | 3.75 | 0.23 | 0.003 |
| 21.19_342.2274m/z | Unknown | 10.52 | 0.87 | 4.02 | 0.23 | 0.003 |
| 20.82_365.8849m/z | Unknown | 0.69 | 0.2 | 0.09 | 0.21 | 0.005 |
| 22.55_342.2274m/z | Unknown | 6.52 | 0.69 | 1.78 | 0.19 | 0.007 |
| 0.99_625.0119m/z | Unknown | 8.19 | 2.2 | 0.75 | 0.18 | 0.005 |
| 19.53_388.2685m/z | Unknown | 1.91 | 0.08 | 0.49 | 0.15 | 0.006 |
| 5.31_729.2220m/z | Unknown | 26.47 | 7.43 | 0.3 | 0.15 | 0.006 |
| 9.42_630.2001m/z | Unknown | 4.33 | 0.75 | 0.51 | 0.15 | 0.005 |
| 7.64_413.1437m/z | Unknown | 7.49 | 2.01 | 0.08 | 0.14 | 0.004 |
| 13.84_314.1968m/z | Unknown | 0.76 | 0.07 | 0.1 | 0.11 | 0.009 |
| 7.42_493.1158m/z | Unknown | 1.68 | 0.12 | 0.18 | 0.09 | 0.005 |
| 7.09_389.1802m/z | Unknown | 5.49 | 0.59 | 0.33 | 0.08 | 0.002 |
| 9.30_748.2449m/z | Unknown | 2.46 | 0.13 | 0.19 | 0.06 | 0.004 |
| 11.62_739.2795m/z | Unknown | 20 | 1.12 | 1.19 | 0.06 | 0.004 |
| 1.24_631.1716m/z | Unknown | 461.62 | 32.29 | 6.65 | 0.04 | 0.005 |
| 13.28_739.2786m/z | Unknown | 6.89 | 0.18 | 0.24 | 0.03 | 0.002 |
