# Supplementary material for: Chorismate mutase and isochorismatase, two potential effectors of the migratory nematode Hirschmanniella oryzae, increase host susceptibility by manipulating secondary metabolite content of rice
Source: Mol Plant Pathol. 2020 Oct 20;21(12):1634–46. doi: 10.1111/mpp.13003 (PMC7694671; doi:10.1111/mpp.13003)
Supplement: Supplementary file 8 — TABLE S4 Differential compounds in the transgenic lines overexpressing HoCM. FC, fold change; FDR, false discovery rate [file MPP-21-1634-s008.docx]

Supplementary table S4: Differential compounds in the transgenic lines overexpressing HoCM. FC: Fold change, FDR: False Discovery Rate.

| **ID** | **Trivial name** | **Mean abundance** | | | | **FC** | **FDR** |
| --- | --- | --- | --- | --- | --- | --- | --- |
|  |  | **CTRL** | **HoCM_FULL2** | **HoCM_CAT2** | **HoCM_CAT3** |  |  |
| 17.10_593.1708m/z | Unknown | 0.05 | 14.57 | 24.36 | 3.17 | 268.3 | 0 |
| 9.74_239.0540m/z | Unknown | 2.25 | 0.45 | 0.28 | 0.46 | 0.18 | 0.007 |
| 1.05_710.9446m/z | Unknown | 81.16 | 8.23 | 12.58 | 15.67 | 0.15 | 0.006 |
| 1.46_429.0338m/z | Unknown | 108.86 | 8.61 | 20.1 | 13.72 | 0.13 | 0.001 |
| 2.24_391.0641m/z | Unknown | 210.71 | 25.73 | 27.25 | 25.67 | 0.12 | 0.01 |
| 7.18_677.1778m/z | Unknown | 29.57 | 2.32 | 4.11 | 3.23 | 0.11 | 0.001 |
| 10.98_791.2370m/z | Unknown | 1.4 | 0.06 | 0.17 | 0.15 | 0.09 | 0.006 |
| 7.82_505.1913m/z | Unknown | 59.76 | 3.91 | 6.87 | 4.32 | 0.08 | 0.001 |
| 10.02_415.1591m/z | Unknown | 1.23 | 0.09 | 0.14 | 0.08 | 0.08 | 0.009 |
| 10.49_415.1595m/z | Unknown | 23.6 | 1.03 | 3.66 | 0.88 | 0.08 | 0 |
| 9.66_377.1447m/z | Unknown | 95.69 | 4.96 | 7.05 | 9.54 | 0.08 | 0.002 |
| 7.84_287.1492m/z | Unknown | 7.56 | 0.37 | 0.28 | 1 | 0.07 | 0.005 |
| 1.53_420.1055m/z | Unknown | 11.78 | 0.31 | 0.89 | 1.17 | 0.07 | 0.007 |
| 1.46_331.2352m/z | Unknown | 21.17 | 0.62 | 2.87 | 0.65 | 0.07 | 0 |
| 8.40_285.1329m/z | Unknown | 2.28 | 0.05 | 0.18 | 0.1 | 0.05 | 0.004 |
| 7.94_373.1488m/z | Unknown | 10.58 | 0.17 | 0.4 | 0.18 | 0.02 | 0 |
| 1.47_663.1399m/z | Unknown | 14.54 | 0.03 | 0.54 | 0.06 | 0.01 | 0 |
