# Supplementary material for: Chorismate mutase and isochorismatase, two potential effectors of the migratory nematode Hirschmanniella oryzae, increase host susceptibility by manipulating secondary metabolite content of rice
Source: Mol Plant Pathol. 2020 Oct 20;21(12):1634–46. doi: 10.1111/mpp.13003 (PMC7694671; doi:10.1111/mpp.13003)
Supplement: Supplementary file 9 — TABLE S5 MS/MS spectral information for a selection of compounds which are differentially abundant in HoCM or HoICM expressing lines. ID, compound identifier; tR, retention time; m/z, mass to charge ratio; Δppm, deviation of measured mass; MS/MS, relative abundances to the base peak of the MS/MS product ions are indicated between parentheses [file MPP-21-1634-s009.docx]

Supplementary table S5: MS/MS spectral info of a selection of compounds which are differentially abundant in HoCM or HoICM expressing lines. ID: compound identifier, tR: retention time, m/z: mass to charge ratio, Δppm: deviation of measured mass, MS/MS: relative abundances to the base peak of the MS/MS product ions are indicated between parentheses.

| ID | trivial name | tR (min) | m/z | formula | Δppm | MS/MS |
| --- | --- | --- | --- | --- | --- | --- |
| 3.26_265.0921m/z | 1-O-ethyl-6-O-glycoloyl hexose | 3.26 | 265.0921 | C10H17O8 | -2.98 | 207.0874 (100) 265.0903 (22) 195.0532 (20) 101.0256 (18) 221.0943 (10) |
| 4.33_309.1185m/z | (1-O-ethyl)hexosyl(6->)ethylene glycol glycolate | 4.33 | 309.1185 | C12H21O9 | -1.96 | 251.1117 (100) 309.12 (21) 207.0873 (7) 101.0256 (1) 233.103 (1) 251.0985 (1) 251.1389 (1) 189.0788 (0.5) 75.0061 (0.5) 61.0308 (0.5) |
|  |  |  |  |  |  |  |
