# Supplementary material for: Chorismate mutase and isochorismatase, two potential effectors of the migratory nematode Hirschmanniella oryzae, increase host susceptibility by manipulating secondary metabolite content of rice
Source: Mol Plant Pathol. 2020 Oct 20;21(12):1634–46. doi: 10.1111/mpp.13003 (PMC7694671; doi:10.1111/mpp.13003)
Supplement: Supplementary file 10 — TABLE S6 Table with primer sequences used in this research [file MPP-21-1634-s010.docx]

Supplementary table S6: Table with primer sequences used in this research.

| **Primer** | **Sequence** | **Goal** |
| --- | --- | --- |
| HoCM-F | ATGTGCAATTTGATTTGCG | Clone full *HoCM* |
| HoCM-R | TCAATTGATGGGAGCACAA |  |
| HoCM_F_noSP | CATTCATCGTCATCCTCCA | Design clone of *HoCM* without signal peptide |
| HoCM_F_CAT | ATGGAAGTGATCCGGCTG | Design clone of *HoCM* with only catalytic domain |
| HoICM_F | ATGGAATTTCTGAAAAAAAGCAA | Clone full *HoICM* |
| HoICM_R | CTATGTGTTAATGTCGTGTCGGA |  |
| attB1HoCM_noSP_F | AAAAAGCAGGCTTCACCATGCATTCATCGTCATCCTCCA | Attach attB sites to different constructs for Gateway cloning |
| attB1HoCM_R | AGAAAGCTGGGTTTCAATTGATGGGAGCACAA |  |
| attB1HoCM_CAT_F | AAAAAGCAGGCTTCACCATGGAAGTGATCCGGCTG |  |
| attB1HoICM_F | AAAAAGCAGGCTTCACCATGGAATTTCTGAAAAAAAGCA |  |
| attB1HoICM_R | AGAAAGCTGGGTTCTATGTGTTAATGTCGTGTCGGA |  |
| attb1 | ACAAGTTTGTACAAAAAAGCAGGCT |  |
| attb2 | ACCACTTTGTACAAGAAAGCTGGGT |  |
| EXP_NARCAI_F | AGGAACATGGAGAAGAACAAGG | expressed protein (LOC_Os07g02340) |
| EXP_NARCAI_R | CAGAGGTGGTGCAGATGAAA |  |
| EIF5C_F | CACGTTACGGTGACACCTTTT | expressed protein (LOC_Os11g21990) |
| EIF5C_R | GACGCTCTCCTTCTTCCTCAG |  |
| 08g08130_1_F | TCCCTCCACCTTTTGATGG | Glutathione S-transferase (LOC_Os08g08130) |
| 08g08130_1_R | TGTAAATAGGCGCACCAACC |  |
| 10g09990_2_F | GGATACGTCTCGGAGTTTTCG | UDP-glycosyltransferase (LOC_Os10g09990) |
| 10g09990_2_R | AAGGCAGGATCTGCTTCG |  |
| 01g04409_2_F | CTTGGTGATGGTGGTTTTGG | serine/threonine-protein kinase (LOC_Os01g04409) |
| 01g04409_2_R | AAGGTTCTGGTGCAGTAGGC |  |
| 01g05610_2_F | AATTCCTCTGTCACTCGTTGG | histone H2B.3 (LOC_Os01g05610) |
| 01g05610_2_R | CTCAGGGCAACCAATTAACC |  |
| 04g52606_2_F | CTTGAGCTCCGTGGAGTAGG | LRR receptor-like serine/threonine-protein kinase (LOC_Os04g52606) |
| 04g52606_2_R | GACACAGTTTGGAAGAACATGC |  |
| 01g02830_2_F | TTTGCATTGTTGGATTGTGG | receptor-like protein kinase (LOC_Os01g02830) |
| 01g02830_2_R | CACAGAAGAATGGCCTTGG |  |
| 07g0417800_2_F | GTTGGTGGATGAACAGTTGG | α-1,2-galactosyltransferase (LOC_Os07g23494) |
| 07g0417800_2_R | ATCTCCCCCTTTGAGCTAGG |  |
| 03g11520_1_F | GGACGTGAGTTCAGTCTCACC | protein RIK (LOC_Os03g11520) |
| 03g11520_1_R | TCGTCTCATCGATGTTGTCC |  |
| 07g09340_2_F | CCCCCATCTATCTTGTGTCC | plasma membrane ATPase (LOC_Os07g09340) |
| 07g09340_2_R | GATTCAGATGGCCAAGAACC |  |
| 07g33954_1_F | GTCATCCTCCTCCCTTTCG | glucose-6-phosphate/phosphate translocator 2 (LOC_Os07g33954) |
| 07g33954_1_R | CGATGCTGAATGTCAGTGG |  |
| QPCR_HoICM_F | TCGATACGATTGCAGTCACC | Check *HoICM* expression |
| QPCR_HoICM_R | ATGCGATAGCCTTTGTCTGC |  |
| QPCR_HoCM_F | GCTGATTGGCAACAGAATGG | Check *HoCM* expression |
| QPCR_HoCM_R | AAGCGAATTGATTGGATTGG |  |
