# Supplementary material for: Chorismate mutase and isochorismatase, two potential effectors of the migratory nematode Hirschmanniella oryzae, increase host susceptibility by manipulating secondary metabolite content of rice
Source: Mol Plant Pathol. 2020 Oct 20;21(12):1634–46. doi: 10.1111/mpp.13003 (PMC7694671; doi:10.1111/mpp.13003)
Supplement: Supplementary file 12 — FILE S2 Detailed protocol describing the metabolomics experiment [file MPP-21-1634-s012.docx]

**Detailed phenolic profiling protocol**

Roots (approximately 5 mg dry weight) were homogenized in liquid nitrogen and extracted with 1 ml methanol. The methanol extract was then evaporated and the pellet dissolved in 200 μl water / cyclohexane (1/1, v/v). 10 μl of the aqueous phase was analyzed via reverse phase UltraHigh Performance Liquid Chromatography (UHPLC; Acquity UPLC Class 1 systems consisting of a Sample Manager-FTN, a Binary Solvent Manager and a Column Manager, Waters Corporation, Milford, MA) coupled to negative ion ElectroSpray Ionization-Quadrupole-Time-of-Flight Mass Spectrometry (ESI-Q-ToF-MS; Vion IMS QTof, Waters Corporation) using an Acquity UPLC BEH C18 column (1.7 μm, 2.1 x 150 mm; Waters Corporation). Using a flow rate of 350 μl/min and a column temperature of 40 °C, a linear gradient was run from 95% aqueous formic acid (0.1%, buffer A) to 50% acetonitrile (0.1% formic acid, buffer B) in 30 min, followed by a concave gradient (curve 3) in 10 min to 100% buffer B. Full MS spectra (m/z 50 – m/z 1,500) were recorded at a scan rate of 10 Hz. The following ESI parameters were used: capillary voltage 2.5 kV, desolvation temperature 550 °C, source temperature 120 °C, desolvation gas 800 L/h and cone gas 50 L/h. Lock correction was applied. In addition to full MS analysis, a pooled sample was subjected to data dependent MS/MS analysis (DDA, exclusion duration = 10 s) using the same separation conditions as above. DDA was performed between m/z 50 and m/z 1,500 at a scan rate of 10 Hz and MS -> MS/MS transition collision energy of 6 eV. The collision energy was ramped from 15 to 30 eV and from 30 to 60 eV for the low and high mass precursor ions, respectively.

Integration and alignment of the m/z features were performed via Progenesis QI software version 2.1 (Waters Corporation). The raw data were imported in this software using a filter strength of 1. A reference chromatogram was manually chosen for the alignment procedure and additional vectors were added in chromatogram regions that were not well aligned. Peak picking was based on all runs with a sensitivity set on ‘automatic’ (value = 5). The normalization was set on ‘external standards’ and was based on the dry weight of the samples. In total, 19,149 m/z features were integrated and aligned across all chromatograms. The subsequent removal of singlet m/z features yielded 18,589 m/z features representing approximately 13,884 compounds based on the m/z feature grouping method described in Morreel et al. (2014). It should be stressed that estimating the number of compounds based on the number of m/z feature groups, yields an upper limit for the number of biological compounds. Nevertheless, contaminants and hetero-adducts between closely co-eluting compounds as well as the rather stringent parameter settings in the m/z feature grouping method will also contribute to an overestimation of the real number of compounds. To handle extreme values, the levels of all m/z features were subjected to an inverse hyperbolic sine transformation (Burbidge et al., 1988) and, whenever necessary, a further Box-Cox transformation was applied to obtain homoscedasticity.

For statistical analysis, the data was split into 2 sets. A first set comprised the two *HoICM* expression lines and the corresponding control line (*HoICM* set). Eight biological replicates were available except for one of the *HoICM* expression lines having only 4 biological replicates. In the second set, one *HoCM_FULL* expression line, two *HoCM_CAT* expression lines and the corresponding control line were included (*HoCM* set). Each line consisted of 8 pooled biological replicates, each pool representing 2 samples. Both sets were subjected to a one-way analysis of variance (ANOVA) using the *lm()* function in R vs 3.4.2 (R_Core_Team, 2017) with a weighing factor included for the first set. For post hoc testing, the *pairwise.t.test(p.adjust=”bonferroni”)* function was applied. In case the data were too heteroscedastic [*bartlett. test()* function, α=0.01], an ANOVA with Welch correction was performed using the *oneway.test(var.equal=F)* function followed by Games-Howell post hoc tests using the *posthocTGH(method=”games-howell”)* function available in the ‘userfriendlyscience’ package (Peters, 2017). The False Discovery Rate (α=0.01) was computed on the ANOVA model significance using the *p.adjust(method=”fdr”)* function. Additional filtering on the prerequisites of (i) a significant post hoc test for each of the transgenic lines and (ii) abundance changes in the same direction for all transgenic lines as compared to the control lines, yielded 54 and 17 differential m/z features (corresponding to 53 and 17 m/z feature groups) for the *HoICM* and *HoCM* sets, respectively.

Structural annotation was performed using a retention time window of 1 min, and both precursor ion and MS/MS identity searches. The precursor ion search (10 ppm tolerance) was based on a compound database constructed via instant JChem (ChemAxon, Budapest, Hungary), whereas MS/MS identities were obtained by matching against an in-house mass spectral database (200 ppm fragment tolerance). As none of the MS/MS spectra of the differential m/z features could be identified via database matching, MS/MS spectral elucidation was attempted (see Supplemental Text) using gas phase fragmentation rules and *in silico* MS/MS elucidation software, i.e. CSI:FingerID (Dührkop et al., 2015, Böcker and Dührkop, 2016) and CFM-ID (Allen et al., 2015).

**MS/MS spectral elucidation**

The compounds eluting at 3.26 min (m/z 265.0921, C_10_H_17_O_8_, Δppm = -2.98) and 4.33 min (m/z 309.1185, C_12_H_21_O_9_, Δppm = -1.96) were both highly accumulating in the HoICM line and their MS/MS spectra were partially showing the same product ions and neutral losses. *In silico* elucidation of both MS/MS spectra via CSI:FingerID (Dührkop et al., 2015) indicated the presence of a hexose moiety in both structures. Both MS/MS spectra were then simultaneously interpreted [see Morreel et al. (2014) for gas phase fragmentation pathways and the references therein; Supp. Fig. 2 and 3]. The CO_2_ loss (product ion at m/z 221.09; Supp. Table 5) upon MS/MS fragmentation of the m/z 265.0921 ion suggested the presence of a carboxylic acid or an ester group. The 58.00-Da neutral losses in both MS/MS spectra (yielding the product ions at m/z 207.09 and m/z 251.11 in the MS/MS spectra of the m/z 265.0921 and m/z 309.1185 precursor ions) represented hydroxyketene (O=C=CH_2_-OH) resulting from the fragmentation of an ester bond. An ester might also charge-remote fragment with the formation of a carboxylate ion; this was evident from the presence of the m/z 75.00 product ion (representing glycolate, HO-CH_2_-COO^-^) in the MS/MS spectrum of the m/z 309.1185 precursor ion. The absence of hexose cross-ring cleavages indicated that the 1-O-position was specifically linked via an acetal function. The mass difference between the hexose derivate of glycolic acid and the compound represented by the m/z 265.0921 ion fits with the mass of ethylene; thus, the m/z 265.0921 ion represents ethylhexoside to which glycolic acid is ester-linked. A putative candidate is 1-O-ethyl-6-O-glycoloyl hexose (smiles: CCOC1OC(COC(=O)CO)C(O)C(O)C1O). The MS/MS product ions for this compound at m/z 207.09, 221.09 and 101.02 were predicted using CFM-ID (Allen et al., 2015).

The mass difference between the ion of 1-O-ethyl-6-O-glycoloyl hexose and the ion of the second compound (m/z 309.1185) represents acetaldehyde (CH_3_-CH=O). The latter was not observed as a neutral loss and is, thus, derived from an internal moiety that was formed by the incorporation of glycol. Therefore, a reasonable structure is (1-O-ethyl)hexosyl(6->)ethylene glycol glycolate (smiles: CCOC1OC(COCCOC(=O)CO)C(O)C(O)C1O). Upon MS/MS spectral prediction by CFM-ID, the product ions at m/z 251.11, 233.10, 207.09, 189.07, 101.03 and 75.00 were confirmed.

ALLEN, F., et al. (2015) Competitive fragmentation modeling of ESI-MS/MS spectra for putative metabolite identification. *Metabolomics,* **11** (1), 98-110.

BÖCKER, S. and DÜHRKOP, K. (2016) Fragmentation trees reloaded. *Journal of cheminformatics,* **8** (1), 5.

BURBIDGE, J. B., et al. (1988) Alternative transformations to handle extreme values of the dependent variable. *Journal of the American Statistical Association,* **83** (401), 123-127.

DÜHRKOP, K., et al. (2015) Searching molecular structure databases with tandem mass spectra using CSI: FingerID. *Proceedings of the National Academy of Sciences,* **112** (41), 12580-12585.

MORREEL, K., et al. (2014) Systematic structural characterization of metabolites in Arabidopsis via candidate substrate-product pair networks. *The Plant Cell,* **26** (3), 929-945.

PETERS, G.-J. (2017) Diamond Plots: a tutorial to introduce a visualisation tool that facilitates interpretation and comparison of multiple sample estimates while respecting their inaccuracy.

R_CORE_TEAM (2017) *R: A Language and Environment for Statistical Computing.* Available from <https://www.R-project.org/>.
